# Regional Characteristics of the Second Wave of SARS-CoV-2 Infections and COVID-19 Deaths in Germany

**DOI:** 10.3390/ijerph182010663

**Published:** 2021-10-12

**Authors:** Gabriele Doblhammer, Daniel Kreft, Constantin Reinke

**Affiliations:** 1 Department of Economics and Social Sciences, Institute for Sociology and Demography, University of Rostock, 18057 Rostock, Germany; daniel.kreft@uni-rostock.de (D.K.); constantin.reinke@uni-rostock.de (C.R.); 2 German Center for Neurodegenerative Diseases, 53127 Bonn, Germany

**Keywords:** machine learning, boosting models, Shap values, mortality, incidence

## Abstract

(1) Background: In the absence of individual level information, the aim of this study was to identify the regional key features explaining SARS-CoV-2 infections and COVID-19 deaths during the upswing of the second wave in Germany. (2) Methods: We used COVID-19 diagnoses and deaths from 1 October to 15 December 2020, on the county-level, differentiating five two-week time periods. For each period, we calculated the age-standardized COVID-19 incidence and death rates on the county level. We trained gradient boosting models to predict the incidence and death rates by 155 indicators and identified the top 20 associations using Shap values. (3) Results: Counties with low socioeconomic status (SES) had higher infection and death rates, as had those with high international migration, a high proportion of foreigners, and a large nursing home population. The importance of these characteristics changed over time. During the period of intense exponential increase in infections, the proportion of the population that voted for the Alternative for Germany (AfD) party in the last federal election was among the top characteristics correlated with high incidence and death rates. (4) Machine learning approaches can reveal regional characteristics that are associated with high rates of infection and mortality.

## 1. Introduction

The second wave of SARS-CoV-2 infections that began in Germany in October 2020 increased exponentially in November, and remained at high levels well into December, despite various regulatory measures beginning in September 2020 and a lockdown beginning in early November 2020. Information about gender, age, and place of residence is the only available information about SARS-CoV-2 infections and COVID-19 deaths, which has hampered a detailed analysis about the drivers of the second wave. However, even during the course of the first wave, there were important factors that were identified both in Germany and internationally.

### 1.1. Social Disparities

There is a consensus that infections and deaths have affected lower social groups the hardest (for a review see [1]) mainly due to their higher mobility during the pandemic and their lower capacity for social distancing [2]. Swedish registry data suggested that the most disadvantaged members of society suffered the most from SARS-CoV-2 infections and had the highest rate of COVID-19 deaths [3].

One of the few German individual-level studies based on health claims data showed that the risk of contracting COVID-19 was highest in occupations where workers had frequent face-to-face contact with COVID-19 patients or potentially infected individuals during their occupational activities. However, increased risks were also observed in occupations with cramped workplaces, suboptimal hygienic conditions, and among persons with temporary employment through agencies [4]. An additional study using health claims data suggested strong social disparities in hospitalizations due to COVID-19 [5].

German ecological studies correlating regional characteristics with SARS-CoV-2 infections and COVID-19 deaths yielded contradictory results. Plümper and Neumayer concluded that only in the second phase of the pandemic and controlling for temporal dependence were predominantly poorer counties affected by COVID-19 [6], whereas Wachtler et al. described a change in the social distribution of infections from regions with high to those with low social status even during the first wave [7]. These studies used a small number of preselected indicators to characterize the regions. A different approach was taken by two studies [8,9] that relied on machine learning algorithms to identify key regional features that predict SARS-CoV-2 infections and COVID-19 deaths without selecting specific indicators. These studies concluded that social status played an important role in addition to features related to geographic location, hotspot events associated with the southern German carnival season, and the proportion of the population at risk in nursing homes. Additionally, they showed that the social gradient had already evolved from a positive to a negative one during the first wave [8].

### 1.2. Ethnic Minorities and Migrants

Studies in the U.S. and U.K. were the first and most prominent to indicate that persons of ethnic minorities were at increased risk for COVID-19 compared with whites (for a systematic review see [10], for U.S., e.g., [11], and U.K., e.g., [12]). A review of clinical outcomes and risk factors for COVID-19 [13] found that migrants were at increased risk of infection and were disproportionately represented among COVID-19 cases and deaths in high-income countries. Comorbidities, barriers related to language, health seeking and health care, cramped housing, risky work, and working conditions [14,15] have been identified as risk factors and vulnerabilities, leading to higher virus exposure. In Germany, migrants are highly represented in occupations with system relevance and thus a higher potential exposition to the virus such as cleaning workers, workers in food production, or nursing of the elderly [16].

### 1.3. Values, Norms and Compliance

While social distancing is essential to contain the spread of COVID-19, not everyone is willing to comply with social distancing measures. From the U.S., there are reports based on debit card transaction data that Democrats were more likely to switch to remote spending after government orders were implemented [17]. Additionally, for the U.S., political conservatism inversely predicted compliance with behaviors aimed at preventing the spread of COVID-19 [18]. For Germany, the “Institute for Democracy and Civil Society” found a correlation of COVID-19 illnesses and deaths with the proportion of voters for the party “Alternative for Germany (AfD)” in the last federal election [19]. Individuals from this party sometimes take prominent roles in protests against corona measures [20]. However, several regions with a high proportion of AfD voters border the neighboring country of the Czech Republic, a hotspot of infection rates, so it is possible that infections have spilled over from there.

### 1.4. Aims

The aim of this study was to identify the key features explaining SARS-CoV-2 infections and COVID-19 deaths during the upswing of the second wave in Germany. Previous studies used partly different indicators and shed light on different factors, so our aim was to consolidate these study results using an overall empirical approach. We did not preselect regional characteristics but instead included a large number of maximally diverse indicators. Using machine learning and a framework for interpreting predictions, we identified the most important features and their associations with regional SARS-CoV-2 infections and COVID-19 deaths.

We designated five two-week periods because incubation time is around 14 days [21,22,23,24,25,26]. Time from symptom onset to death also depends on the health care system with a median of 11 days in the first wave in Germany [27] and 18 days in an international study [28]. We began with low infection numbers from 1–15 October 2020, and continued with the period 16–31 October 2020, when the exponential increase in infections developed. The third period extended from 1–15 November 2020, and the fourth from 16–30 November 2020. In both periods, the exponential increase continued. The final period, from 1–15 December 2020, was characterized by a continued increase in infections. Infections reached a peak around Christmas time, which was outside our study period. During these five periods, a number of regulatory measures were taken to combat increasing SARS-CoV-2 infections [29,30]. From 1 August 2020, anyone entering Germany from abroad could be tested for SARS-CoV-2 free of charge; from 4 September 2020, anyone entering Germany from a risk area had to be quarantined for at least 10 days.

Since 29 September 2020, the hotspot strategy has been used to respond to the pandemic in a two-step, region-specific manner. For example, private parties could be held in public spaces with a maximum of 50 people if 35 new infections per 100,000 population were detected within seven days. From 50 new infections per 100,000 inhabitants within seven days, private parties in public spaces could only take place with a maximum of 25 people.

Beginning 15 October 2020, people in patient-centered settings such as hospitals or nursing homes were tested for SARS-CoV-2 infections with rapid antigen tests. Employees, visitors, and residents or persons under care were required to be tested.

On 3 November 2020, a partial lockdown began. People were asked to refrain from private parties altogether; to limit private gatherings with friends, relatives, and acquaintances to a fixed wider household; to refrain from recreational activities and visits to areas frequented by the public as well as from nonessential private travel and tourist day trips; to refrain from nonessential stays in enclosed areas frequented by the public or nonessential travel on public transportation; and to make visits, especially to the elderly and vulnerable, only if all family members were free of symptoms of the disease and have not been exposed to any particular risk in the previous days. Schools and daycare centers as well as stores remained open; restaurants, bars, clubs, discotheques, and pubs were closed. On 25 November 2020, the partial closure was extended until 20 December 2020, and stricter regulations were imposed on larger stores and shopping centers. From 15 December 2020, all at-risk groups had access to free or discounted filtering face piece respirators (FFP2 masks).

## 2. Data and Methods

### 2.1. Data

We downloaded data (26 January 2021) from the Robert Koch Institute [31], which provides information on COVID-19 diagnoses and deaths by sex, age (age groups: 0–4, 5–14, 15–34, 35–59, 60–79, 80+), and 401 counties (NUTS3 region). Only cases with nucleic acid detection or pathogen isolation are published in the data hub [32]. Individual patients were not involved in this study.

Population size on the county level was derived from the DESTATIS regional database at the end of the year 2019 [33]. Because age is a known risk factor for SARS-CoV-2 infections and COVID-19 deaths and can lead to multicollinearity problems with county characteristics, we directly calculated age-standardized incidence and death rates on the county level to control for differences in age distribution. We used the German age distribution from the year 2019 from the Regional Database of the Statistical Offices of the Federation and the Länder (2021).

### 2.2. Selection of the Characteristics

Given the literature reviewed above, we selected macro variables in nine domains (number of indicators in brackets). SES-socioeconomic status (61); urbanity/density (22); health (20); care need (7); regional connectedness (15); norms and values (3); special geographic location (11); population composition in terms of foreigners/people with migration background (4); ageing; and (age) structure of the population (12). All regional macro factors are of high quality because they were collected and harmonized by administrative institutions and are based on established and reliable data sources and measurements. The individual indicators consider a variation of different aspects of the dimensions. The selection criteria were comparability, variability between regions, and multidimensionality with respect to the different indicators.

The data stemmed from the INKAR (Indikatoren und Karten zur Raum- und Stadtentwicklung) database (2020) of the Federal Institute for Research on Building, Urban Affairs and Spatial Development (BBSR) [34]; latitude and longitude were defined in terms of the centers of the county capitals. The proportion of Catholics stemmed from the 2011 census, the emission data on particulate matter with a diameter of 10 micrometers (µm) or less (PM10) from the German Environment Agency Database (UBA), main diagnoses in hospitals by place of residence in 2017 from the Regional Database of the Statistical Offices of the Federation and the Länder [35], and the international COVID-19 incidence rates from the European Center for Disease Prevention and Control [36]. See data availability statement below for access to the data and Table S1 in the Supplementary Materials for the list of independent variables and their descriptive values. All 155 indicators were numeric or dummies taking the values zero or one.

### 2.3. Analysis Strategy

**Figure 1:**
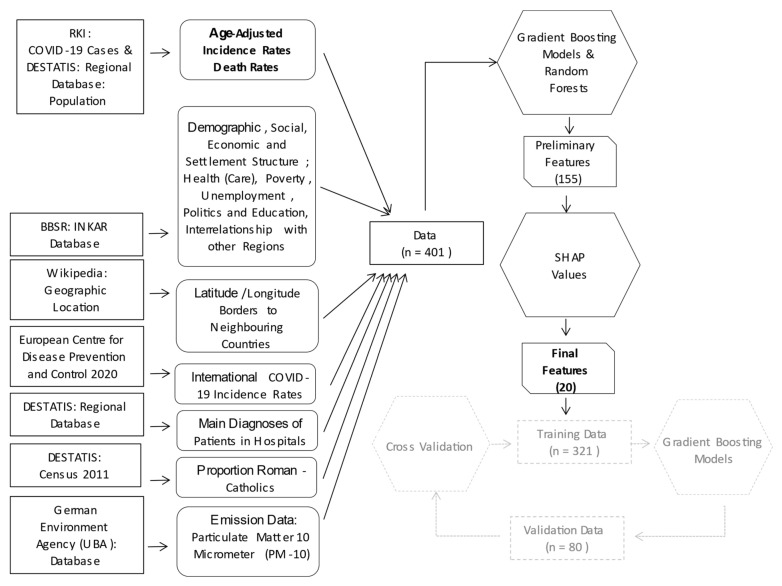
Analysis strategy.

Our analysis strategy consisted of three steps: First, we trained gradient boosting models to predict the age-standardized incidence and death rates for each period with the 155 characteristics of the counties; these characteristics are termed features (Figure 1) and consist of all variables of the nine domains described above. The models also included the previous period’s age-standardized incidence rates to account for the presence of infections. For counties bordering neighboring countries, the previous period’s age-standardized incidence in the neighboring country was also included. Gradient boosting models were trained using the CatBoostRegressor from the CatBoost algorithm [37]. As an alternative, we used the random forest regressor from the Scikit-learn module in Python [38] with 5000 trees. We kept all other hyperparameters at their default values. We calculated the R^2^ and root mean squared (RMSE) errors to evaluate how well the models fit the data. Second, we used Shap values to explore the importance of the features and third, we characterized the 20 most prominent features in terms of negative/positive correlations with each of the two outcome variables.

The Shap value is the average contribution of a feature value to the prediction in different combinations of all feature values. The higher the average contribution, the more important the feature. In addition, the Shap value provides the correlation of the feature with the outcome variable [39]. We used the SHAP (SHapley Additive exPlanations) procedure [40] and display the Shap values in plots in the Supplementary Materials. We provide the ranking of the features according to their average contribution in the results section below.

We categorized the top 20 associations identified by the Shap values into twelve categories depicting the correlation between the feature and the outcome: 1 = positive SES gradient (SES high): higher incidence rates in high SES regions; 2 = negative SES gradient (SES low): higher incidence in low SES regions; 3 = urban/high density gradient (urban): higher incidence in urban/high density regions; 4 = rural/low density gradient (rural): higher incidence in rural/low density regions; 5 = care need gradient: higher incidence associated with high care need; 6 = health gradient: higher incidence associated with poor health; 7 = community’s connectedness low (connect low): higher incidence associated with low connectedness; 8 = community’s connectedness high (connect high): higher incidence associated with high connectedness; 9 = international migration high (migration high): higher incidence associated with higher proportion of foreigner/people with migration background; 10 = geography; 11 = values and norms; 12 = age/aging structure of the region. For example, if the characteristic “median household income” is positively correlated with the infection/death rate, then this is categorized as a positive SES gradient; if the correlation is negative, then this is called a negative SES gradient. We counted the number of the top 20 features in each category and interpreted the evolution of their frequency over time as well as the ranking of each individual feature.

As a sensitivity analysis, we repeated step one and fit a second model for each period using only the 20 most prominent features identified by the first model. We calculated R^2^ and RMSE to evaluate how much variance in the age-standardized incidence and death rates are covered by these 20 features.

To evaluate the out-of-sample model performance, we applied k-fold random subsampling [41] using 20 folds. For each period, we split the data at random to fit a model on a training set (80%) using the 20 most prominent features. This model was used to make predictions on a test set (20%) and to calculate the RMSE. Then, a linear regression model was applied to explain the predictions by the actual response values from the test set. R^2^ from the linear regression model indicated how much variance from the actual response values can be explained by the predictions.

In an additional sensitivity analysis, we identified all characteristics with pairwise correlations smaller/larger than −0.8/+0.8 and excluded the characteristics that were more highly correlated with all other characteristics. In a third sensitivity analysis, a geographical distance matrix of all geometric centers of the individual regions to each other was calculated using the free GeoDa software package on the basis of the maps with the 2019 boundaries. The first-order Queen method was chosen as the distance matrix method (i.e., the distances of all directly adjacent regions in all cardinal directions were considered). Spatial lag regression models (SLR-models) were calculated for the top 20 features with longitude and latitude and for the top 10 features without longitude and latitude. The features were neither standardized nor mean-centered, which means that the coefficients obtained depend on the scaling of the original features. All analyses were performed using Python 3.8.3. (Python Software Foundation, Wilmington, DE, USA) and Stata 16.0. (StataCorp LLC, College Station, TX, USA).

## 3. Results

### 3.1. Age Standardized COVID-19 Incidence and Death Rates in the Five Periods

Age-standardized incidence rates (Table 1) quadrupled from the first half of October (1–15 October: 52.45 per 100,000) to the second half (196.86), reaching 300 cases in the second half of November and 360 in the first half of December.

Age-standardized death rates (Table 1) were still low in early October (1–15 October: 0.50 new cases per 100,000), then doubled almost every two weeks from mid-October onward (16–31 October: 2.46) and reached a maximum in December (1–15 December: 11.66).

These growth curves showed distinct geographic patterns that changed over time, as shown in Supplementary Figures S1–S5. Both incidence and mortality moved from west to east, starting in the high-incidence regions of North Rhine-Westphalia, Baden-Württemberg, and Bavaria, and then moving to Saxony, Thuringia, and western Bavaria.

### 3.2. Model Fitting and Diagnostics

With few exceptions, boosting models tended to outperform random forests in terms of accuracy, hence below we discuss the results of the boosting models and present the random forests in the Supplementary Materials. First, we compared the models based on all features with those based on the twenty most important features. Then, we discuss the twenty most important features.

Using all features resulted in R^2^ values of 0.99 and above, using only the subset of features resulted in almost unchanged R^2^ values, but increased RMSE values. This indicates that the boosting algorithm produced well-fitted models, even when only a subset of the most prominent features was used. Out-of-sample performance increased across periods, with the performance of the models for death rates always well below that of incidence rates (incidence rate: R^2^ = 0.4911 in the first period to 0.7428 in the last period; death rate: R^2^ = 0.1722 in the first period to R^2^ = 0.4213 in the last period). Boosting models and random forests are all in Supplementary Table S2).

### 3.3. Model Results

The age-standardized incidence and death rates changed over time, as did the key features. Taking the first twenty features according to their Shap values (Period 1: Figure S6a,b; Period 2: Figure S7a,b; Period 3: Figure S8a,b; Period 4: Figure S9a,b; Period 5: Figure S10a,b in the Supplementary Materials), we grouped them into the categories outlined above. We counted the number of features in each category and found that features related to SES, urbanity/density, and health were present in all time periods; those representing the connectedness of a region were present in the period from mid-October to mid-November and again in December. Features related to need for care started to show up in the second half of November and in December, while those related to migration were present in October and the first half of November. Features reflecting values and norms were present in all periods, as did those characterizing the (age) structure and aging process in a region (Supplementary Table S4).

#### 3.3.1. SES

**Figure 2:**
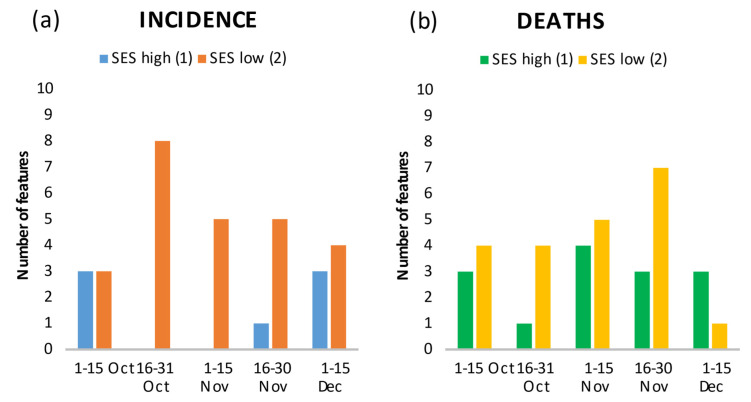
Number of features in the top 20 showing the relationship between low and high SES, and incidence (**a**) and death rates (**b**) by time period.

At the beginning of the second wave (1–15 October), the overall low incidence was comparatively elevated in both high and low SES regions (Figure 2a: of the first 20 features, three were associated with low SES and three with high SES); with the exponential increase in infections, low SES regions were more heavily affected; no single feature was associated with high SES (Figure 2a: 16–31 October (eight of 20 features related to low SES); 1–15 November (five of 20 features related to low SES). During the peak period (16–30 November; 1–15 December), infections again spilled over from low to high SES regions (Figure 2a).

COVID-19 deaths were correlated with both low and high SES regions in all periods, with generally higher correlation with low SES regions (Figure 2b).

Of note, at the beginning of the second wave, the features that indicated a positive correlation of infections and deaths with high SES regions ranked ahead of those that were correlated with low SES regions. This changed in the second period. For example, in the second period, the top ranking SES features for both infections and deaths indicated a negative gradient (infections rank 6: persons_no_qualification = “%Persons without any professional qualification in all employed persons in 2017”; mortality rank 2: Change_long_unemployment_rate = “%Change in long-term unemployment rate in 2012–2017”) (Supplementary Figure S7a,b).

#### 3.3.2. Migration and Foreigners

**Figure 3:**
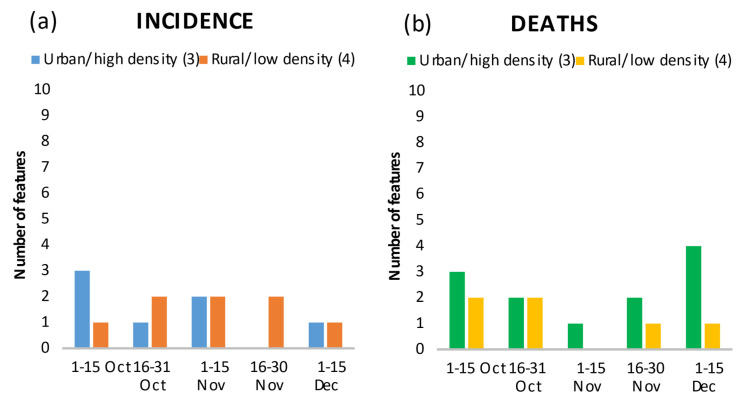
Number of features in the top 20 showing the relationship between migration and geographic dispersion, and incidence (**a**) and death rates (**b**) by time period.

Features related to international migration background were among the top characteristics through mid-to-late November and correlated positively with incidence (Figure 3a) and death rates (Figure 3b). In the first period, the regional “%Foreigners in the total population in 2017” (Foreigners_in_total_population) ranked third in incidence and tenth in mortality. In the second period, it still ranked fourth in incidence and eleventh in mortality, and in the third period, it ranked sixth in mortality (Supplementary Figures S6–S8).

#### 3.3.3. Geography, Urbanity/Rurality, Connectedness

**Figure 4:**
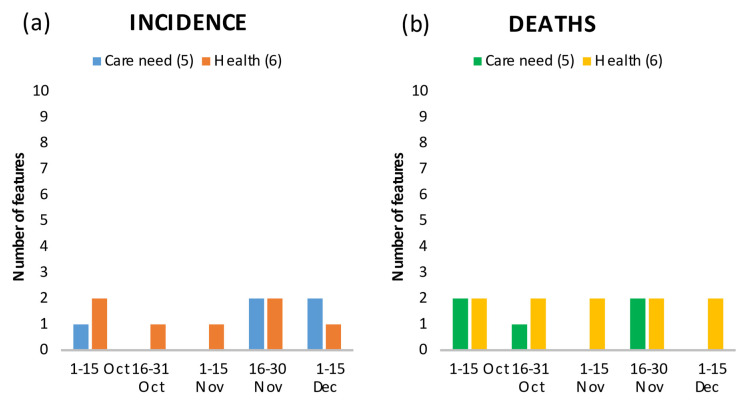
Number of features in the top 20 showing the relationship between urbanity/rurality and density, and incidence (**a**) and death rates (**b**) by time period.

**Figure 5:**
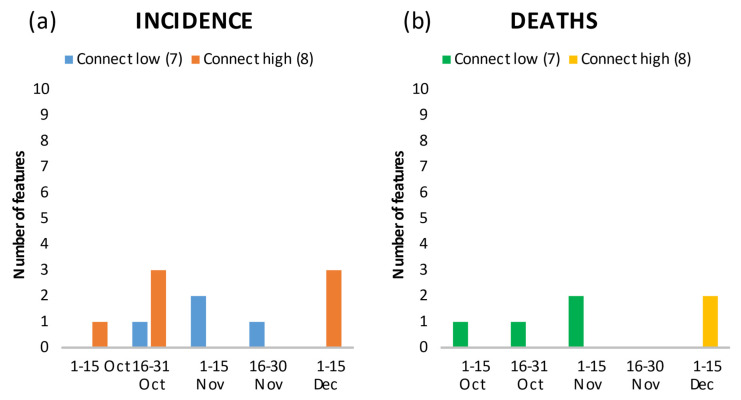
Number of features in the top 20 showing the relationship between connectedness, and incidence (**a**) and death rates (**b**) by time period.

Geography in the form of latitude and longitude, which can be considered a residual category in characterizing the disease spread, was among the top features in all periods (Figure 3a,b; longitude and latitude in Supplementary Figures S6–S10). In the second period, the incidence of the previous period (Inc_previoud_period_289) and “Federal border with Austria” (Border_Austria) and their positive correlation with incidence (Supplementary Figure S7) may indicate a spill-over effect of the high incidence in Austria to Germany. Geographical spread was independent of urbanity/rurality (Figure 4a,b) and connectivity of a region (Figure 5a,b). Over the periods, infections and deaths occurred in both urban and rural regions, and in better or less connected regions. It is interesting to note the positive correlation of the second ranked feature “Nitrogen surplus per agricultural area in kg/ha in 2016” (Nitrogen_surplus) and the fourth rank of the feature “particulate matter with a diameter of 10 micrometers (µm) or less” (PM_10) with deaths in the first period (Supplementary Figure S6).

#### 3.3.4. Care Need and Health

**Figure 6:**
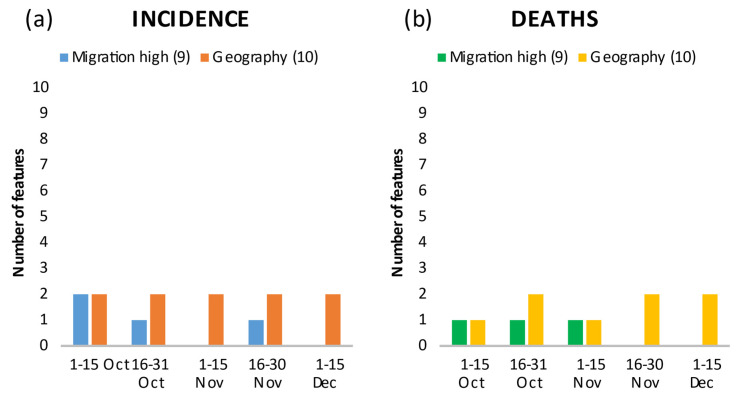
Number of features in the top 20 showing the relationship between care need and health, and incidence (**a**) and death rates (**b**) by time period.

The proportion of the population in need of long-term care is another important characteristic, as suggested by its presence among the top 20 in all periods (Figure 6a,b). In the first period, the feature “%Persons in long-term inpatient care out of all persons in need of care” (Persons_inpatient_long_care) was among the top 20, but was negatively correlated with incidence and death rates, suggesting fewer infections and lower mortality for regions with a high proportion of the population in need of care living in nursing homes. This changed from the second period onwards, where regions with a large inpatient long-term care sector or a large proportion of people in need of care living in nursing homes had higher death rates (e.g., Period 4 positive correlation: Stuff_nursing_home and Persons_inpatient_long_care) and regions with a large outpatient long-term care sector had lower death rates (e.g., Period 4 negative correlation: Stuff_outpatient_services) (Supplementary Figures S6, S7 and S9).

Features representing population health (Figure 6a,b) were generally negatively correlated with incidence and death rates. The highest-ranking feature in all periods was the incidence of SARS-CoV-2 infections in the previous period (Inc_previous_period) (Supplementary Figures S6–S10). However, there was also a positive correlation between infections and deaths, and the proportion of persons with a diagnosis of endocrine, nutritional, and metabolic diseases (Rate_endocrine_diseases), which includes diabetes (periods 3 and 4, Supplementary Figures S8 and S9).

#### 3.3.5. Values and Norms, and Age/Ageing

**Figure 7:**
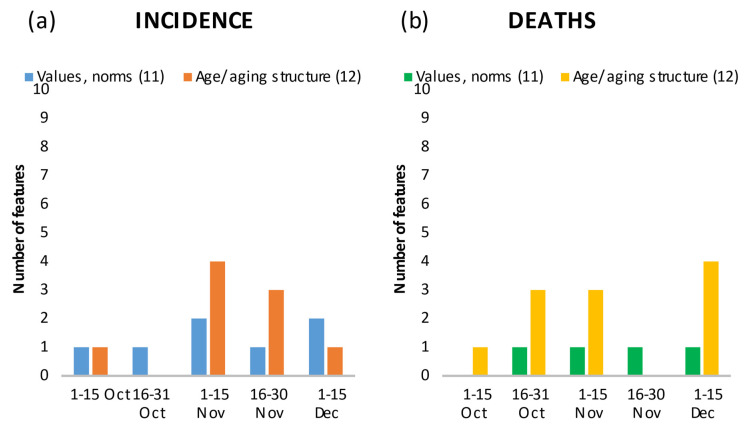
Number of features in the top 20 showing the relationship between values/norms and age/ageing structure, and incidence (**a**) and death rates (**b**) by time period.

Of the features that depict values and norms (Figure 7a,b), one consistently appeared in the top 20, was positively correlated with incidence and death rates, and increased in feature importance over the course of the wave. In the second period, the feature “%Valid votes for AfD out of all valid votes in 2017” (Valid_votes_for_AfD) ranked thirteenth (deaths only); in the third period, it ranked eighth (incidence) and seventh (deaths); in the fourth period, it ranked third (incidence) and second (deaths), and in the fifth period, it ranked third (incidence) and fifth (deaths) (Supplementary Figures S6–S10). Finally, population characteristics related to age and (aging) structure (Figure 7a,b) played an important role with decreasing importance for incidence, but an increasing one for deaths over the periods.

#### 3.3.6. Sensitivity Analyses

Our first sensitivity analysis using random forests revealed similar features among the top 20 (Supplementary Figures S11–S15). Our second sensitivity analysis excluded one of the pairwise highly correlated features by excluding the feature that itself had higher correlations with other features. The results did not change. Our third sensitivity analysis, which estimated associations between the top 20 features and incidence/deaths based on SLR models, found similar associations to the Shap values. However, because some of the features were not statistically significant including latitude and longitude, we reran the SLR models for the top 10 features without latitude and longitude. The models accounting for spatial proximity to surrounding regions and the coefficients of the features confirmed the direction of the associations based on the Shap values (Supplementary Tables S5–S14).

## 4. Discussion

Combining an ecological study design with machine learning techniques using 155 county-level regional variables, we examined potential associations between regional characteristics and SARS-CoV-2 infections and COVID-19 deaths. This study design helped us to avoid imposing our expectations on the pre-selection of possible regional characteristics. We restricted our analysis to the period between early October and mid-December 2020, defined as the second-wave upswing [30,42], and divided it into five two-week periods. These periods reflect the exponentially increasing infections that peaked in mid-December, followed by increases in deaths.

### 4.1. SES

Restricting our analysis to those first twenty risk factors identified by Shap values, we concluded that, similar to the first wave [6,8,9], SES characteristics of a region was an important factor in the second wave. While both social gradients, positive and negative, were present in SARS-CoV-2 infections in October, the negative SES gradient began to dominate over time and was always the dominant one in mortality. Higher mobility of high SES groups during periods of low infection and a greater decrease in mobility in periods of high infection may explain this trend. U.S. studies showed that the poorest areas moved from the lowest to highest mobility and thus had fewer opportunities for social distancing [2]. Another explanation may lie in the particular nature of inequalities in work-related health and economic risks as they apply to workers in the service and manufacturing sectors in Germany [43].

### 4.2. Migration/Foreigners and Borders with Neighboring Countries

International migration and a high proportion of foreigners living in a county were important regional characteristics during those periods when the exponential increase in incidence intensified. On one hand, international contacts in these regions may have been higher and more infections may have been imported from abroad. For the beginning of the exponential increase (second half of October), we found higher infection rates for districts where neighboring countries had high infection rates, especially for those bordering Austria. On the other hand, a high proportion of foreigners may be indicative of a negative social gradient in infections and deaths, as they often work in occupations such as cleaners, food production workers, or elderly care [16,44], and are therefore more exposed to the virus. A high proportion of foreigners may also be related to the presence of different cultures and norms [45], and lack of access to information about the pandemic [46], leading to differences in adherence to protective measures such as wearing masks and maintaining social distance. On one hand, results from the UCL COVID-19 Social Study suggested that working outside the home, as common for migrants in low-wage employment in Germany [16,44] was associated with lower compliance [47]. On the other hand, in Switzerland, non-compliance with COVID-19-related public health measures was higher among young adults without a migration background [48].

### 4.3. Nursing Home Population and Health

In our study, characteristics of a region related to the nursing home population and care provided by outpatient care services remained among the top features explaining high versus low rates of infection and death. This was despite the fact that, compared with the first wave, the proportion of COVID-19 deaths in nursing homes decreased as a proportion of all deaths [49]. The high importance of these features reflects the sad fact that this particularly vulnerable group is difficult to protect [50] (for an international review about protection measures see [51]), despite commonly applied, but also highly criticized isolation measures [52,53]. In addition, there was evidence that COVID-19 outbreaks in nursing homes were associated with spatial deprivation and that the latter was a major risk factor for COVID-19 deaths in nursing home residents [54].

Population health appeared to be another important characteristic, which was associated with a negative SES gradient and influenced SARS-CoV-2 infections and COVID-19 deaths. We included information on regional health profiles reflecting known comorbidities of severe COVID-19 cases [55]. Incidence and death rates were found to be higher in regions with a high proportion of diagnosed endocrine, nutritional, and metabolic diseases. This includes diabetes mellitus, which has been repeatedly identified as a major risk factor for severe COVID-19 outcomes and mortality [56].

### 4.4. Values/Norms and Compliance

One feature that became more important during the second wave was the proportion of the population that voted for the party “Alternative for Germany (AfD)” in the last federal election. In the exponential growth phase, this was the third most important characteristic of a region in terms of infections and the second most important in terms of deaths. Although this correlation has been noted before, it has also been highly controversial [19]. We consider this characteristic as a possible indicator of compliance. Numerous surveys have suggested that COVID-19 is a deeply partisan issue in the U.S., and that partisanship was more strongly associated with physical disengagement than numerous other factors including county SARS-CoV-2 infections, population density, median income, and racial and age demographics [57]. Studies in Germany showed that respondents from eastern Germany and those with little trust in public institutions were particularly critical of containment measures [58]. In a sensitivity analysis, we also included the proportion of votes for other parties, which did not affect the importance of the feature related to AfD votes. In contrast, a second feature was additionally identified, namely the residual category all other parties, consisting of individuals who vote for non-establishment parties from the left to right spectrum and are therefore more critical of the mainstream. This feature was positively correlated with incidence rates. The “Deutschlandtrend” survey of the public broadcaster “ARD” found clear preferences among voters of the parties represented in the Bundestag. In mid-May 2020, 61 percent of AfD supporters were in favor of lifting containment measures, compared with 25 to 34 percent among supporters of the ruling coalition parties CDU/CSU and SPD [59]. In Germany, this preference goes hand in hand with a lack of trust in public institutions [60].

Note that the feature “%Roman-Catholics” in a county, which was prominent in the first wave [8], lost importance in the second wave. In the first wave, it reflected large gatherings during the carnival season in southern Germany, which led to hotspots of infection [61]. During the second wave, large gatherings occurred mainly during demonstrations against Corona measures, and SARS-CoV-2 infection rates were particularly high in regions of the protesters’ origin after these demonstrations [62].

### 4.5. Urbanity/Rurality and Connectedness

Similar to the first wave, there was no pronounced urban/high density gradient or gradient associated with the connectedness of a region [8]. Population density per se did not appear to be a risk factor, which is supported by a regional analysis of COVID-19 prevalence in the United States [63] and Germany [9]. Cities have both the healthiest and unhealthiest populations. The former benefit from better infrastructure and access to health care, while the latter have a higher burden of disease and lower life expectancy due to behavioral and environmental risk factors [64].

### 4.6. Study Limitations

Our study is hampered by a series of limitations. Reliance on the county level introduces the problem of the modifiable areal unit [65]. County-level data might be too coarse, but also too finely graded to detect important features driving the pandemic. To overcome the limitation that the macro variables were restricted to Germany, we included the age standardized incidence in neighboring countries for counties with international borders.

True infection rates for SARS-CoV-2 cannot be discerned due to asymptomatic individuals, regional approval criteria for testing that resulted in different testing rates, and differences in reporting by local health departments to the RKI. In addition, these data report the time of diagnosis rather than the time of infection. There was also a strong weekday effect, with lower reporting rates on weekends. Our 14-day period averages these different lags and yields a more straightforward picture of infections. In addition, our models included information about infections in the previous period.

The RKI included data in the data hub only when infections were confirmed by nucleic acid detection or pathogen isolation. In these patients, the causes of death in the majority of those who died appeared to be directly related to COVID-19 and were not a direct consequence of preexisting health conditions and comorbidities [66,67]. However, it is important to consider that beyond COVID-19-related mortality, non-COVID-19-related mortality may also increase due to delayed treatment and hospitalization as well as deficits in monitoring and care of dependent individuals [68]. In Germany, a significant increase in cardiovascular mortality was observed while catherization activity was reduced [69].

Different machine learning algorithms identify different features and their importance. We obtained similar results regardless of the machine learning algorithm used: random forests versus cat boosting algorithms, with the latter better reflecting the data with few exceptions. Nevertheless, it is important to keep in mind that the Shap values explain the model and not the data. However, the SLR models estimated in our sensitivity analysis supported the conclusions derived from the Shap values.

## 5. Conclusions

Our study showed that an ecological approach using explainable machine learning methods can help shed more light on the regional infection patterns of COVID-19 in Germany. Ecological analyses have their place in stimulating innovation in a rapidly evolving field of research [70] where individual data are not available yet. Although ecological analysis cannot provide insights into the mechanisms and does not allow for inference regarding individuals, it can highlight potential drivers. Our study showed that a number of regional characteristics were crucial for the increase in infections in the second wave. As in the first wave, they moved from high to low SES regions. Risky working conditions with reduced opportunities for social distancing, a high burden of chronic disease, and residence in nursing homes may underlie this concentration in low-SES regions. In addition, regional patterns of voting behavior were associated with infections and deaths, possibly indicating norms and values associated with non-adherence to Corona measures. To further elucidate these findings, we urgently need more individual-level data [71].

## Figures and Tables

**Table 1:** Distribution of age-standardized COVID-19 incidence and death rates per 100,000 person-years by period (*n* = 401 counties, IQR interquartile range).

Period	Mean	SD	Min	10%	25%	50%	75%	90%	Max	IQR
	Incidence rate per 100,000 population (age-standardized)									
1–15 Oct.	52.45	36.79	3.43	15.07	26.03	44.78	67.13	104.67	267.67	41.10
16–31 Oct.	196.86	100.08	21.63	82.17	124.02	180.95	250.00	331.38	613.03	125.98
1–15 Nov.	294.75	132.15	51.45	128.84	187.58	295.15	375.24	456.30	734.39	187.66
16–30 Nov.	299.44	144.25	20.30	135.46	203.50	286.18	371.91	481.42	968.59	168.41
1–15 Dec.	360.45	194.99	35.50	176.19	245.80	322.59	428.30	564.44	1239.70	182.49
	Death rate per 100,000 population (age-standardized)									
1–15 Oct.	0.50	0.89	0.00	0.00	0.00	0.00	0.74	1.55	4.68	0.74
16–31 Oct.	2.46	2.77	0.00	0.00	0.48	1.70	3.26	6.18	15.95	2.78
1–15 Nov.	4.85	4.09	0.00	0.81	1.91	3.78	6.79	10.34	29.78	4.88
16–30 Nov.	7.96	7.88	0.00	1.24	3.02	6.11	10.70	15.69	64.76	7.68
1–15 Dec.	11.66	9.76	0.00	2.67	5.24	8.94	15.34	24.98	68.45	10.10

## Data Availability

The following datasets were derived from sources in the public domain: Robert Koch Institute, ESRI. RKI COVID19. dl-de/by-2-0 (https://npgeo-corona-npgeo-de.hub.arcgis.com/datasets, accessed on 26 January 2021). Statistical Offices of the Federation and the Länder Regional database (https://www.regionalstatistik.de/genesis, accessed on 27 January 2021). DESTATIS Census 2011: Census database (https://ergebnisse.zensus2011.de, accessed on 6 April 2021). INKAR Database: Federal Institute for Research on Building, Urban Affairs, and Spatial Development. INKAR—Indikatoren und Karten zur Raum- und Stadtentwicklung 2020 (https://www.inkar.de/, accessed on 25 November 2020). European Center for Disease Prevention and Control: Download historical data (to 14 December 2020) on the daily number of new reported COVID-19 cases and deaths worldwide (https://www.ecdc.europa.eu/en/publications-data/download-todays-data-geographic-distribution-covid-19-cases-worldwide, accessed on 29 January 2021) The following data are available on request from the data holder: Emission data: German Environment Agency Database (UAB): https://www.umweltbundesamt.de/en (received on 22 May 2019).

## Supplementary Materials

The following are available online at https://www.mdpi.com/article/10.3390/ijerph182010663/s1, Figure S1: Period 1—Regional distribution of age-standardized COVID-19 incidence and death rates, Figure S2: Period 2—Regional distribution of age-standardized COVID-19 incidence and death rates, Figure S3: Period 3—Regional distribution of age-standardized COVID-19 incidence and death rates, Figure S4: Period 4—Regional distribution of age-standardized COVID-19 incidence and death rates, Figure S5: Period 5—Regional distribution of age-standardized COVID-19 incidence and death rates, Figure S6: Period 1—SHAP summary plots of the first twenty features (a) age-standardized incidence, (b) age-standardized death rates, Figure S7: Period 2—SHAP summary plot of the first twenty features (a) age-standardized incidence, (b) age-standardized death rates, Figure S8: Period 3—SHAP summary plot of the first twenty features (a) age-standardized incidence, (b) age-standardized death rates, Figure S9: Period 4—SHAP summary plot of the first twenty features (a) age-standardized incidence, (b) age-standardized death rates, Figure S10: Period 5—SHAP summary plot of the first twenty features (a) age-standardized incidence, (b) age-standardized death rates, Table S1: List of independent variables and descriptives, Table S2: R2 and RMSE scores of boosting models for all periods (Incidence), Table S3: R2 and RMSE scores of boosting models for all periods (death rates), Table S4: Number of features in the top 10/20 features indicating a positive correlation with high COVID-19 incidence and death rates.
