# Supplementary material for: Regional Characteristics of the Second Wave of SARS-CoV-2 Infections and COVID-19 Deaths in Germany
Source: Int J Environ Res Public Health. 2021 Oct 12;18(20):10663. doi: 10.3390/ijerph182010663 (PMC8535595; doi:10.3390/ijerph182010663)
Supplement: Supplementary file 1 [file ijerph-18-10663-s001.zip › ijerph-1377036-supplementary.pdf]

Supplementary Table S1: List of independent variables and descriptives

Variable name	Label	Category	Mean (sd)
Area_in_natural_state	%Area in natural state in total area in 2017	Urbanity/density	5.1 (3.71)
Area_natural_state_sqm	Area in natural state in sqm per person in 2017	Urbanity/density	2.9 (3.52)
Area_population_100km	Area size-weighted population in a distance of 100 km in 1,000 persons in 2017	Urbanity/density	33367.32 (30681.78)
Average_household_income	Average household income per person in 2016	SES	1591.69 (589.47)
Beds_in_hospitals	Beds in hospitals per 1000 persons in 2016	Health	6.35 (3.89)
Beds_nursing_homes	Beds in nursing homes per 100,000 persons in 2017	Care need	113.19 (28.86)
Border_Austria	Border with Austria	Geography	0.04 (0.2)
Border_Belgium	Border with Belgium	Geography	0.01 (0.11)
Border_Czech_Republic	Border with Czech Republic	Geography	0.04 (0.21)
Border_Denmark	Border with Denmark	Geography	0.01 (0.09)
Border_France	Border with France	Geography	0.05 (0.21)
Border_Luxemburg	Border with Luxemburg	Geography	0.01 (0.1)
Border_Netherlands	Border with The Netherlands	Geography	0.03 (0.18)
Border_Poland	Border to Poland	Geography	0.02 (0.15)
Border_Switzerland	Border with Switzerland	Geography	0.01 (0.11)

Care_receivers_long_care	%Care allowance receivers in all persons in long-term care in 2017	Care need	50.86 (6.52)
Cars_persons	Cars per 1,000 persons in 2017	Connectedness	579.16 (70.98)
Change_average_household_income	%Change in average household income per person in 2012-2016	SES	8.95 (2.96)
Change_beds_hospitals	%Change in beds in hospitals in 2012-2016	Health	-0.67 (9.1)
Change_employed_primary_sector	%Change in employed persons in primary sector in all employed persons in 2012-2016	SES	-6.16 (22.53)
Change_employed_secondary_sector	%Change in employed persons in secondary sector in all employed persons in 2012-2016	SES	1.13 (5.36)
Change_employed_tertiary_sector	%Change in employed persons in tertiary sector in all employed persons in 2012-2016	SES	3.8 (3.63)
Change_gross_income	%Change of gross income per employee in 2012-2016	SES	14.99 (4.46)
Change_gross_income_industry	%Change in gross income per employee in production industry in 2012-2016	SES	11.95 (7.73)
Change_gross_value_added	%Change in gross value added in 2012-2016	SES	13.64 (5.68)
Change_life_expectancy_at_birth	%Change of life expectancy at birth in 1993/94/95 and 2015/2016/2017	Health	7.49 (1.18)
Change_long_unemployment_rate	%Change of long-term unemployment rate in 2012-2017	SES	-8.35 (18.69)

Change_marginally_employed	%Change of marginally employed persons in 2012-2017	SES	1.27 (7.73)
Change_marginally_employed_older	%Change of number of marginally employed persons 65+ in 2012-2017	SES	34.56 (13.28)
Change_number_births	%Change of number of births in 1995-2017	Age/Aging	16.51 (8.78)
Change_number_marriages	%Change of number of marriages in 2012-2017	Age/Aging	5.23 (9.95)
Change_number_persons	%Change of number of persons in 2012-2017	Age/Aging	2.21 (2.78)
Change_number_persons_age_50to65	%Change of number of persons at age 50-65 in 2012-2017	Age/Aging	9.54 (4.67)
Change_number_persons_age_65to75	%Change of number of persons at age 65-75 in 2012-2017	Age/Aging	-3.95 (4.8)
Change_out_commuters_300km	%Change of outbound commuters over a distance of 300km+ in all employed persons in 2007-2017	Connectedness	0.02 (0.55)
Change_out_commuters_50km	%Change of outbound commuters over a distance of 50km+ in all employed persons in 2007-2017	Connectedness	0.24 (1.41)
Change_persons_age6to18_overall	%Change of persons aged 6 to <18 in all persons in 2012-2017	Age/Aging	-1.1 (7.79)
Change_persons_younger6_overall	%Change of persons aged 5 and younger in all persons in 2012-2017	Age/Aging	11.58 (4.92)
Change_proportion_foreigners	%Change of proportion of foreigners in 2012-2017	Foreigners/Migration	3.33 (1.11)
Change_proportion_young_employed	%Change of proportion of young employed persons (under 26 years) in all employed in 2011-2017	SES	5.67 (9.24)
Change_share_older_employed	%Change of share of older employed persons in all employed persons in 2011-2017	SES	38.3 (7.72)

Change_tourist_beds	%Change in beds in tourist facilities per 1,000 persons in 2012-2017	SES	-86.78 (6.36)
Change_unemployment_rate_older	%Change in older age unemployment rate (55 years+) in 2012-2017	SES	-.34 (15.46)
Change_unemployment_rate_younger	%Change in younger age unemployment rate (under 26 years) in 2012-2017	SES	-9.04 (22.95)
Debts_core_households	Debts of the core households in " per person in 2016	SES	1682.7 (1549.75)
distance_next_pharmacy	Average distance to the next pharmacy (population-weighted in straight-line) in 2017	Urbanity/density	1418.31 (808.55)
distance_next_station	Average distance to the next stop/station (population-weighted in straight-line) in 2017	Connectedness	531.12 (495.52)
distance_next_supermarket	Average distance to the next supermarket (population-weighted in straight-line) in 2017	Urbanity/density	1050.97 (542.18)
Employed_academic_degree	%Employed persons with academic degree in all dependently employed persons in 2017	SES	13.06 (6.2)
Employed_academic_degree30to35	%Employed persons at age 30-35 with academic degree in all dependently employed in 2017	SES	2.1 (1.45)
Employed_crafts_sector	Employed persons in crafts sector per 100 employable persons in 2017	SES	13.99 (4.89)
Employed_finance_housing	%Employed persons in finance and housing in all employed in service sector in 2016	SES	22.24 (4.54)
Employed_industry_employable	Employed persons in industry per 100 employable persons in 2017	SES	18.25 (8.72)

Employed_primary_sector1	%Employed persons in primary sector in all dependently employed persons in 2017	SES	1.14 (1.27)
Employed_primary_sector2	%Employed persons in primary sector in all employed persons in 2016	SES	2.07 (1.79)
Employed_producing1	%Employed persons in producing professions in all employed persons in 2016	SES	68.93 (11.2)
Employed_producing2	%Employed persons in producing professions in all dependently employed persons in 2016	SES	30.27 (7.12)
Employed_region	Employed persons per sqm of region in 2016	Urbanity/density	327.22 (491.33)
Employed_secondary_sector1	%Employed persons in secondary sector in all dependently employed persons in 2017	SES	31.87 (11.46)
Employed_service_sector1	Employed persons in service sector per 100 employable persons in 2017	SES	39.24 (14.84)
Employed_without_qualification	%Employed persons without qualification in all dependently employed persons in 2017	SES	11.65 (3.18)
Foreigners_in_total_population	%Foreigners in total population in 2017	Foreigners/Migration	10.03 (5.15)
Forest_area	Forest area in sqm per person in 2017	Urbanity/density	1886.79 (1906.89)
GDP_per_employee	GDP in 1,000" per employed person in 2016	SES	67.05 (11.97)
GDP_per_person	GDP in 1,000" per person in 2016	SES	35.61 (15.81)

General_practitioner	General practitioner per 100,000 persons in 2017	Health	61.36 (26.11)
Graduates_higher_qualification	%Graduates with higher education entrance qualification in all graduates in 2017	SES	32.46 (8.86)
Graduates_secondary_degree	%Graduates with secondary education degree in all graduates in 2017	SES	16.57 (5.04)
Gross_income_per_employee	Gross income per employee in 2016	SES	2337.79 (806.78)
Gross_income_production_industry	Gross income per employee in production industry in 2016	SES	3039.73 (1074.39)
Gross_value_added	Gross value added in total in 1,000" per employed person in 2016	SES	60.39 (10.78)
High_school_students	%High school students in all pupils in 2017	SES	26.36 (6.69)
Households_average_income	%Households with average income (1,500" - 3,600" per month) in all households in 2016	SES	48.2 (1.58)
Households_high_income	%Households with high income (>3,600" per month) in all households in 2016	SES	21.17 (5.41)
In_commuters_employees	%Inbound commuters in all employed persons in 2017	Connectedness	64.51 (10)
Inc_previous_period_274	Cum. incidence per 100.000 for 14 days in neighbour country at day 274	Health	20.84 (44.33)
Inc_previous_period_274	Age-standardized incidence rate per 100,000 person-years from 16.09. to 30.09.2020	Health	26.81 (19.82)

Inc_previous_period_289	Cum. incidence per 100.000 for 14 days in neighbour country at day 289	Health	78.52 (176.94)
Inc_previous_period_289	Age-standardized incidence rate per 100,000 person-years from 01.10. to 15.10.2020	Health	52.45 (36.79)
Inc_previous_period_305	Cum. incidence per 100.000 for 14 days in neighbour country at day 305	Health	193.53 (426.17)
Inc_previous_period_305	Age-standardized incidence rate per 100.000 person-years from 16.10. to 31.10.2020	Health	196.86 (100.08)
Inc_previous_period_320	Cum. incidence per 100.000 for 14 days in neighbour country at day 320	Health	201.51 (400.71)
Inc_previous_period_320	Age-standardized incidence rate per 100,000 person-years from 01.11. to 15.11.2020	Health	294.75 (132.15)
Inc_previous_period_335	Cum. incidence per 100.000 for 14 days in neighbour country at day 335	Health	127.61 (265.31)
Inc_previous_period_335	Age-standardized incidence rate per 100,000 person-years from 16.11. to 30.11.2020	Health	299.44 (144.25)
International_net_migration	International net migration per 1,000 persons in 2017	Foreigners/Migration	4.81 (6.63)
Latitude	Latitude	Geography	50.62 (1.74)
Life_expectancy_at_birth	Life expectancy at birth in 2017	Health	80.66 (1.01)
Long_unemployed	%Long-term unemployed persons (unemployed for one year and longer) in 2017	SES	32.2 (8.08)

Longitude	Longitude	Geography	9.87 (2.03)
Marginally_employed_older1	%Marginally employed persons in all persons 65+ in 2017	SES	12.9 (2.77)
Marginally_employed_older2	%Marginally employed persons 65+ in all marginally employed persons in 2017	SES	15.15 (3.23)
max_1km_to_pharmacy	%Persons with max. 1km distance to pharmacy in 2017	Urbanity/density	61.76 (18.47)
max_1km_to_station	%Persons with max. 1km distance to stop/station in 2017	Connectedness	88.7 (14.05)
max_1km_to_supermarket	%Persons with max. 1km distance to supermarket in 2017	Urbanity/density	69.28 (15.82)
Median_wages_full_employee	Median wages of full-time dependently employed persons in 2017	SES	2834.29 (882.01)
Mwages_full_employed_25to55	Median wages of full-time dependently employed persons at age 25-55 in 2017	SES	2846.08 (926.15)
Net_commuters	%Net sum of commuters in all employed persons in 2017	Connectedness	-10.36 (29.72)
Nitrogen_surplus	Nitrogen surplus per agricultural area in kg/ha in 2016	Urbanity/density	68.33 (27.16)
Old_age_dependency_ratio	Old-age (65+) dependency ratio in 2017	Age/Aging	34.35 (5.47)
Older_employed_employed	%Older employed persons (55 years+) in all employed persons in 2017	SES	20.19 (2.24)

Older_employed_older	%Older employed persons in all older persons (55 years+) in 2011-2017	SES	52.53 (3.6)
Older_unemployed_unemployed	%Older unemployed (55 years+) in all unemployed persons in 2017	SES	22.97 (4.19)
Open_area	%Open area (incl. water and agricultural areas) in total area in 2017	Urbanity/density	81.22 (12.82)
Out_commuters_150km	%Outbound commuters over a distance of 150km+ in all employed persons in 2017	Connectedness	4.41 (1.35)
Out_commuters_300km	%Outbound commuters over a distance of 300km+ in all employed persons in 2017	Connectedness	2.4 (.89)
Out_commuters_50km	%Outbound commuters over a distance of 50km+ in all employed persons in 2017	Connectedness	11.48 (3.48)
Persons_academic_qualification	%Persons with an academic qualification in all employed persons in 2017	SES	11.96 (5.17)
Persons_aged_6to18_overall	%Persons aged 6 to <18 in all persons in 2017	Age/Aging	10.8 (0.93)
Persons_basic_social_benefits	%Persons with basic social security benefits per 1,000 persons in 2017	SES	9 (4.15)
Persons_density_under150	%Persons in municipalities with a population density <150 inh/sqkm in 2017	Urbanity/density	29.51 (30.14)
Persons_employees_sqkm	Persons and employees per sqkm in 2017	Urbanity/density	727.79 (1056.47)
Persons_in_long_care	Persons in long-term care per 10.000 persons in 2017	Care need	428.13 (106.03)

Persons_inpatient_long_care	%Persons in inpatient long-term care in all persons in long-term care in 2017	Care need	24.36 (5.37)
Persons_no_qualification	%Persons without any professional qualification in all employed persons in 2017	SES	11.71 (3.01)
Persons_older65_social_benefits	%Persons at age 65+ with basic social security benefits in persons aged 65+ in 2017	SES	2.62 (1.53)
Persons_outpatient_long_care	%Persons in outpatient long-term care in all persons in long-term care in 2017	Care need	23.82 (5.23)
Persons_per_sqkm	Persons per sqkm in 2017	Urbanity/density	490.63 (677.72)
Persons_qualification_employed	%Persons with a professional qualification in all employed persons in 2017	SES	66.92 (6.63)
Persons_settlement_traffic	%Persons per sqkm settlement and traffic area in 2017	Urbanity/density	1828.86 (1057.55)
Persons_short_job_employed	%Persons with a short-time job in all employed persons in 2017	SES	0.75 (1.44)
Persons_younger6_overall	%Persons aged 5 and younger in all persons in 2017	Age/Aging	5.37 (0.46)
Pharmacies	Pharmacies per 100,000 persons in 2017	Urbanity/density	27 (4.9)
PM_10	particulate matter with a diameter of 10 micrometers (µm) or less	Urbanity/density	14.25 (2.2)
Premature_mortality	Premature mortality (deaths of persons younger than 65 years) per 1,000 persons in 2017	Health	1.72 (.36)
Private_debitors	Private debitors per 100 persons in 2017	SES	9.69 (2.73)

Pupils_in_grade_11	%Pupils in grade 11 in all pupils in 2017	SES	3.81 (1.14)
Rate_circulatory_system_diseases	Age standardized rate of I00-I99 of diseases of the circulatory system per 10,000 persons in 2017	Health	350.95 (70.65)
Rate_endocrine_diseases	Age standardized rate E00-E99 of Endocrine, nutritional and metabolic diseases per 10,000 persons in 2017	Health	64.05 (15.09)
Rate_respiratory_system_diseases	Age standardized rate of J00-J99 of diseases of the respiratory system per 10,000 persons in 2017	Health	156.74 (33.15)
Recreational_area	%Recreational area in total area in 2017	Urbanity/density	2.49 (2.68)
Recreational_area_in_sqm	Recreational area in sqm per person in 2017	Urbanity/density	70.51 (56.73)
Remaining_life_expectancy_age_60	Remaining life expectancy at age 60 in 2017	Health	23.7 (.66)
Roman_catholics	%Roman-catholics in 2011	Norms and values	32.24 (24.36)
School_leavers_without_degree	%School leavers without any degree in 2017	SES	6.56 (2.24)
Sex_ratio_age_20to40	Sex ratio (females to males) at age 20-40 in 2017	Age/Aging	0.92 (0.05)
Stuff_nursing_homes	Stuff in nursing homes per 10,000 persons in 2017	Care need	97.71 (23.28)
Stuff_out_patient_services	Stuff in care services per 10,000 persons in 2017	Care need	47.1 (18.7)
time_next_highway	Average travel time to the next highway with car in minutes in 2018	Connectedness	11.86 (8.37)
time_next_international_airport	Average travel time to the next international airport with car in minutes in 2018	Connectedness	49.62 (21.98)

time_next_large_regional_center	Average travel time to the next large-sized regional center ("Oberzentrum") in 2018	Connectedness	22.56 (16.03)
time_next_medium_regional_center	Average travel time to the next medium-sized regional center ("Mittelzentrum") in 2017	Connectedness	6.79 (5.55)
time_next_national_train_station	Average travel time to the next national train station with car in minutes in 2017	Connectedness	21.93 (15.38)
Total_fertility_rate	Total fertility rate in 2017	Age/Aging	1.63 (0.13)
Total_net_migration	Total net migration per 1,000 persons in 2017	Foreigners/Migration	5.1 (4.28)
Tourist_beds	Beds in tourist facilities per 1,000 persons in 2017	SES	41.78 (49.31)
Traffic_accidents_persons	Traffic accidents per 100,000 persons in 2017	Urbanity/density	491.09 (87.67)
Unemployment_rate_young	Unemployment rate of young persons (under 26 years) in 2017	SES	5.26 (2.71)
Valid_votes_for_AfD	%Valid votes for AfD in all valid votes in 2017	Norms and values	13.39 (5.33)
Voter_turnout	%Voter turnout (Number of valid votes in the last Bundestag election) of all registered voters in 2017	Norms and values	75.08 (3.79)
Water_area	%Water area in total area in 2017	Urbanity/density	2.4 (2.8)
Water_area_in	Water area in sqm per person in 2017	Urbanity/density	123.13 (181.27)
Young_employed_employed	%Young employed persons (under 26 years) in all employed persons in 2017	SES	20.86 (2.79)

Young_employed_young	%Young employed persons in all young persons (under 26 years) in 2017	SES	49.56 (5.94)
Young_unemployed_unemployed	%Young unemployed (under 26 years) in all unemployed persons in 2017	SES	9.83 (2.02)

Supplementary Table S2: R^2 and RMSE scores of boosting models for all periods (Incidence)

	1st Oct - 15th Oct	16th Oct - 31th Oct	1st Nov - 15th Nov	16th Nov - 30th Nov	1st Dec - 15th Dec
R^2 first model (all features)	0.9994	0.9994	0.9995	0.9993	0.9996
RMSE first model (all features)	0.8771	2.4047	2.9688	3.7350	3.7249
R^2 second model (20 features)	0.9971	0.9963	0.9976	0.9972	0.9983
RMSE second model (20 features)	1.9621	6.0657	6.4013	7.6349	8.0680
R^2 second model (out-of-sample)	0.4911	0.6369	0.7247	0.7108	0.7428
RMSE second model (out-of-sample)	26.7168	61.2432	72.8262	79.6925	93.5302

Supplementary Table S3: R^2 and RMSE scores of boosting models for all periods (death rates)

	1st Oct - 15th Oct	16th Oct - 31th Oct	1st Nov - 15th Nov	16th Nov - 30th Nov	1st Dec - 15th Dec
R^2 first model (all features)	0.9990	0.9974	0.9987	0.9987	0.9989
RMSE first model (all features)	0.0279	0.1408	0.1404	0.2841	0.3221
R^2 second model (20 features)	0.9954	0.9918	0.9915	0.9946	0.9949
RMSE second model (20 features)	0.0598	0.2500	0.3753	0.5752	0.6944
R^2 second model (out-of-sample)	0.1722	0.1814	0.2773	0.3038	0.4213
RMSE second model (out-of-sample)	0.7927	2.6000	3.6652	6.0477	6.9774

Supplementary Figure S1: Period 1 - Regional distribution of age-standardized COVID-19 incidence and death rates

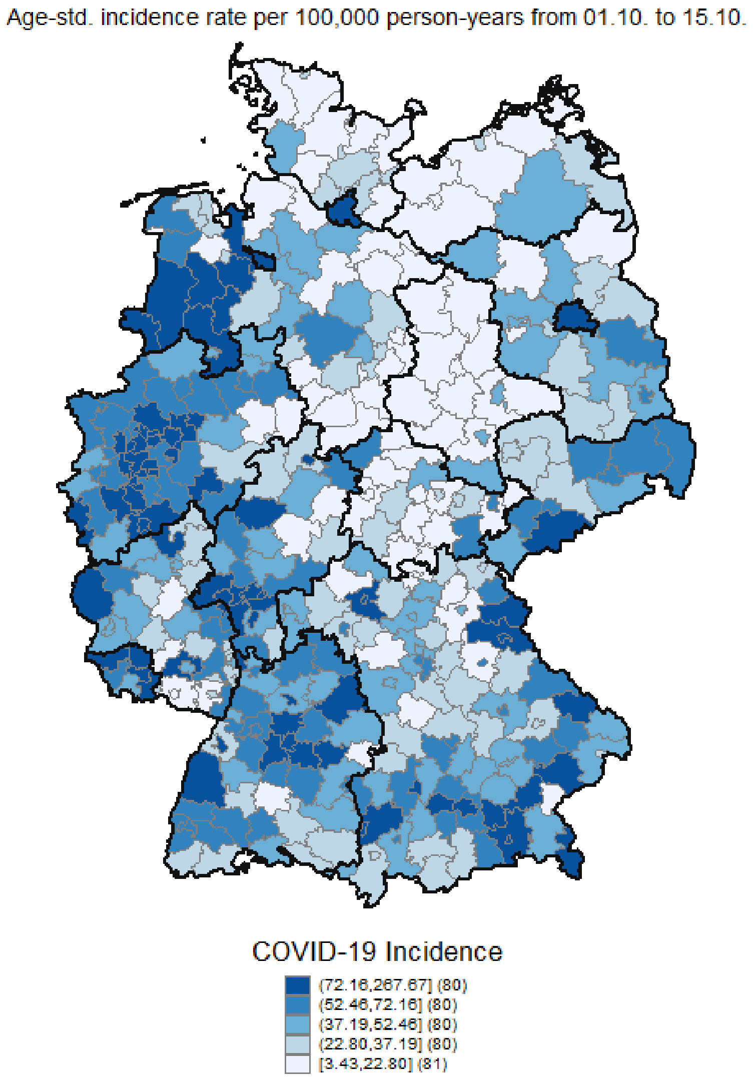

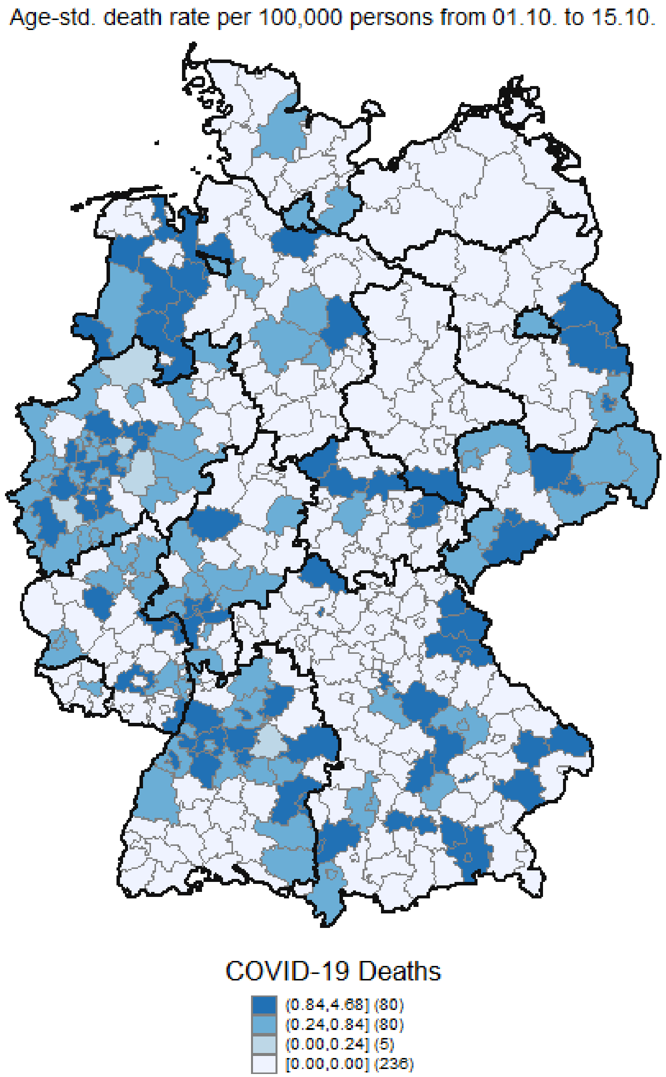

Supplementary Figure S2: Period 2 - Regional distribution of age-standardized COVID-19 incidence and death rates

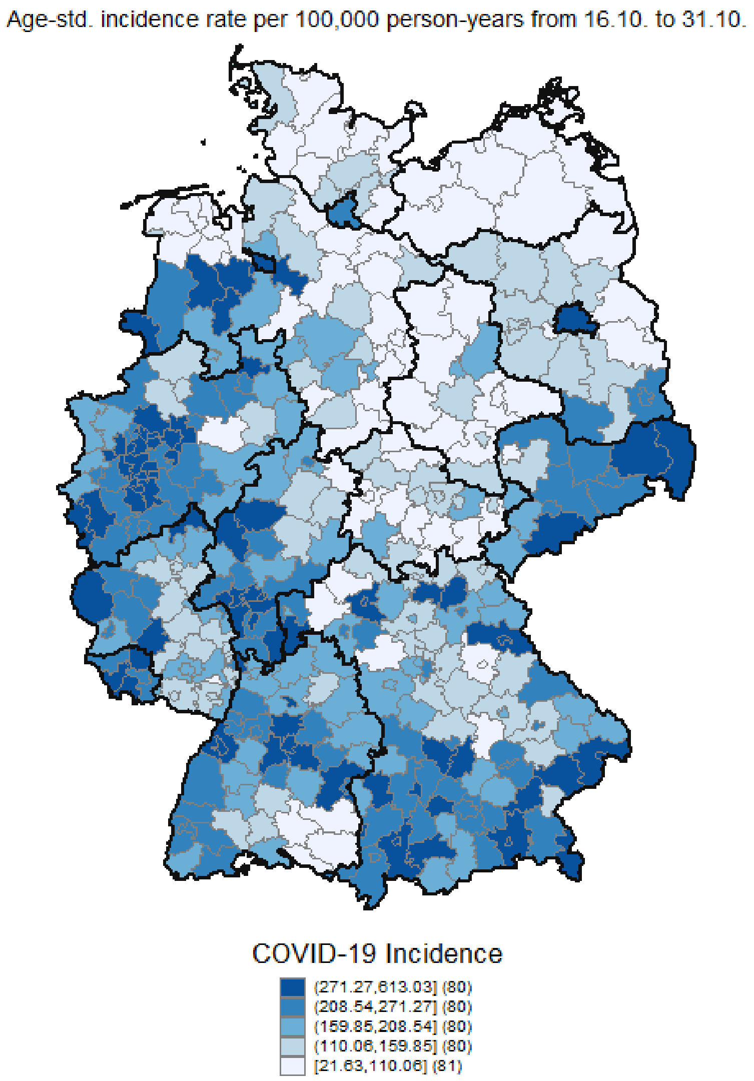

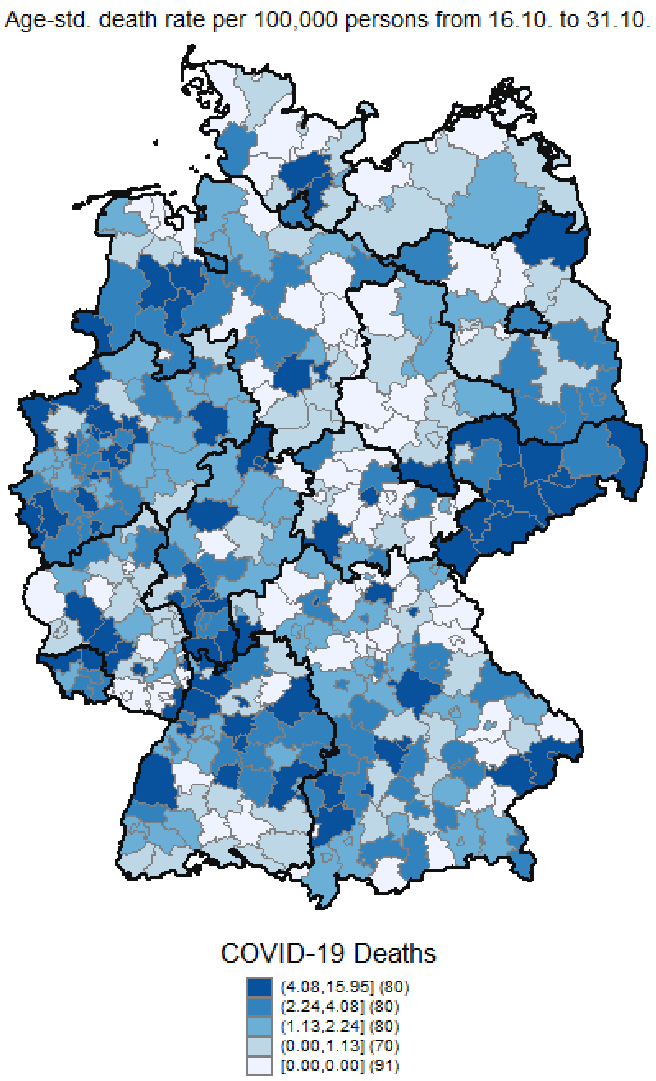

Supplementary Figure S3: Period 3 - Regional distribution of age-standardized COVID-19 incidence and death rates

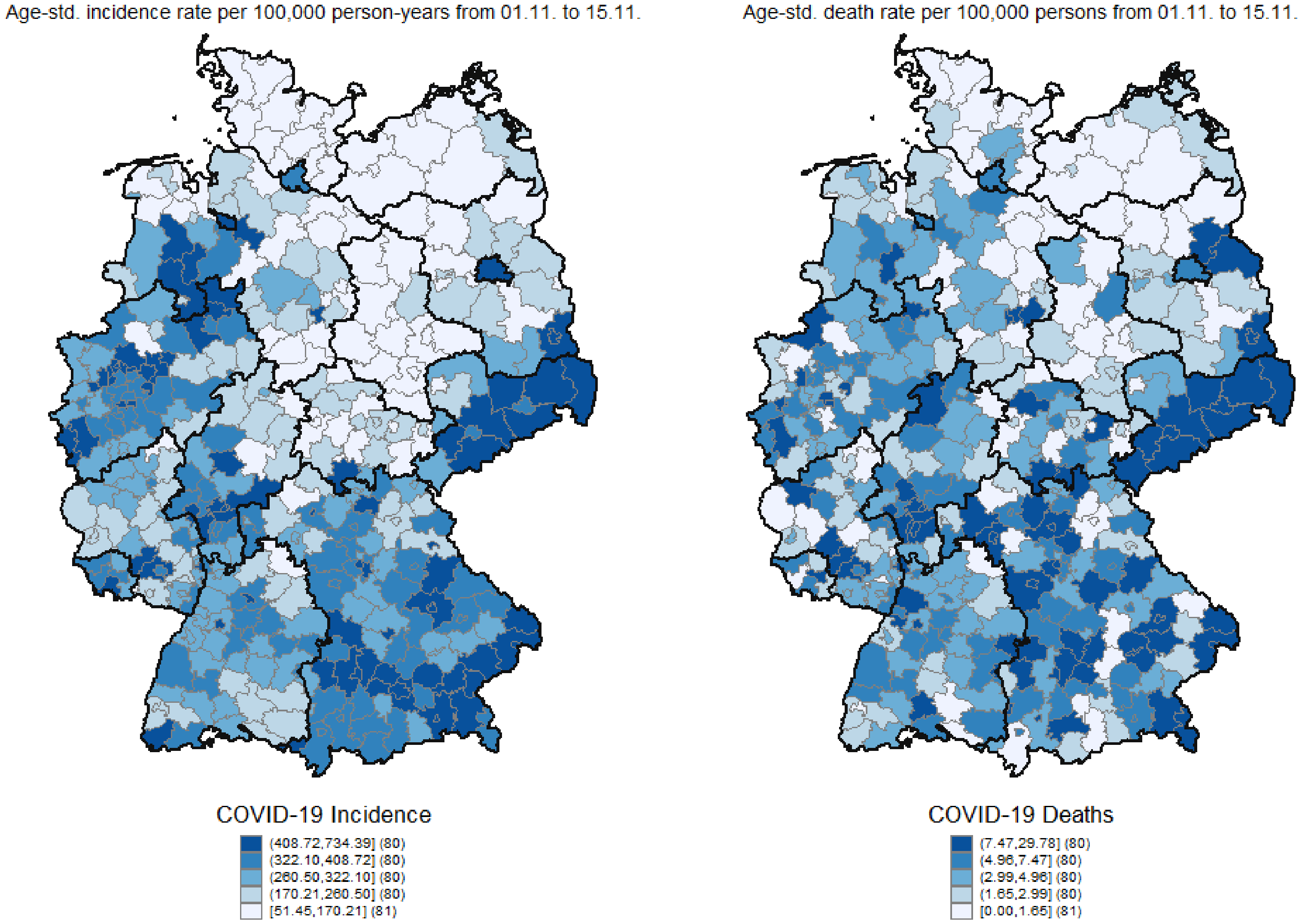

Supplementary Figure S4: Period 4 - Regional distribution of age-standardized COVID-19 incidence and death rates

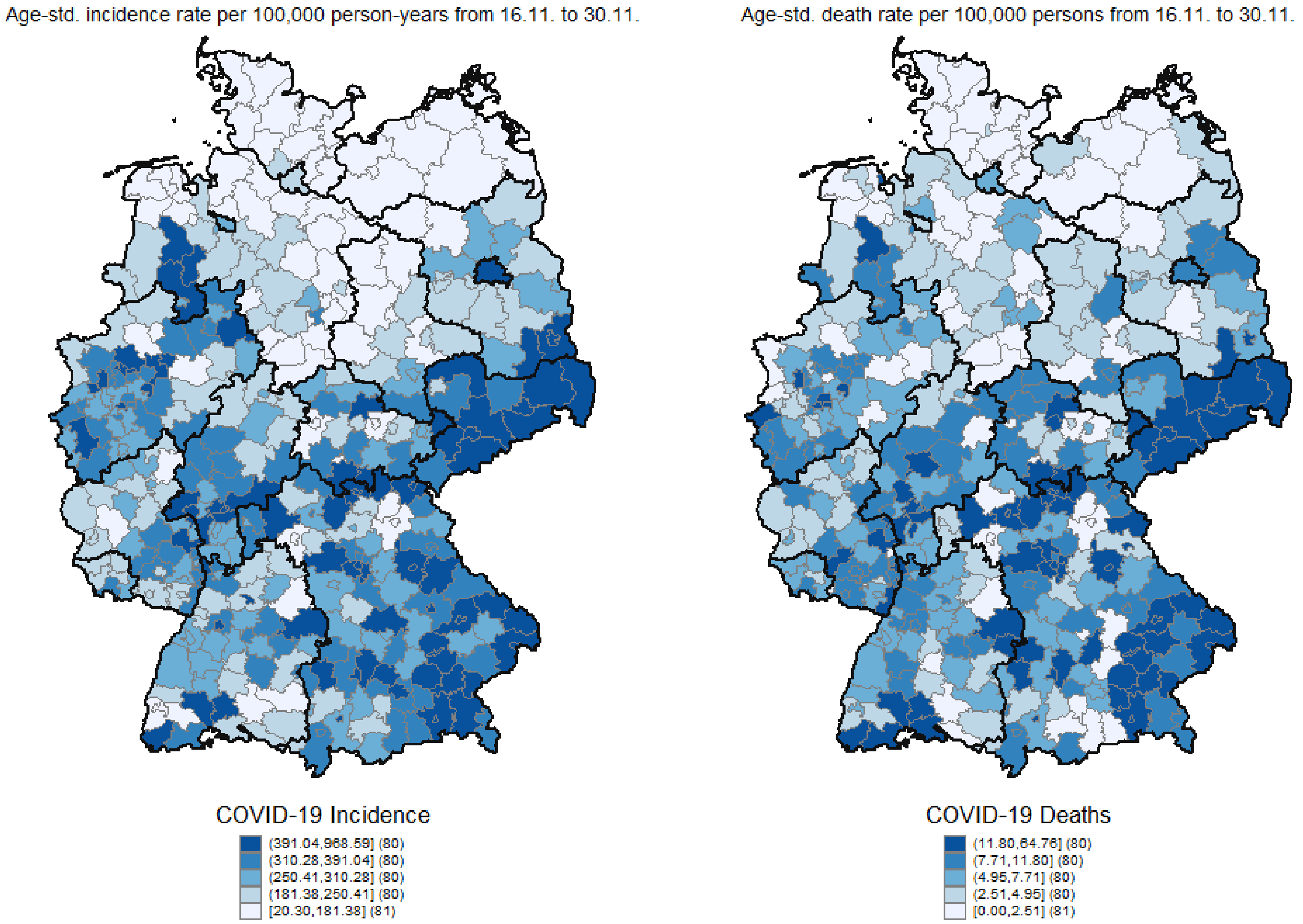

Supplementary Figure S5: Period 5 - Regional distribution of age-standardized COVID-19 incidence and death rates

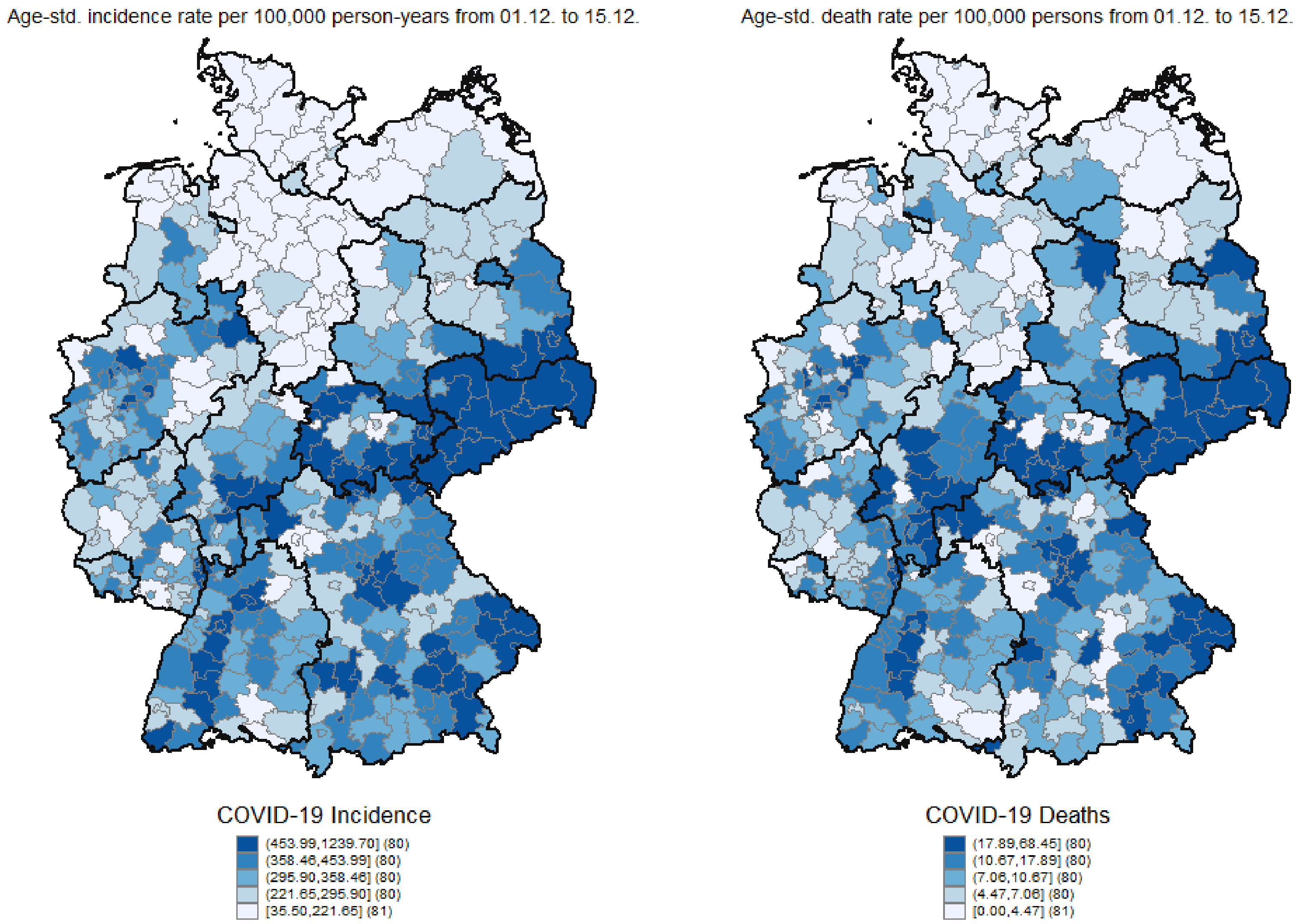

Supplementary Figure S6: Period 1 - SHAP summary plots of the first twenty features (a) age-standardized incidence, (b) age-standardized death rates

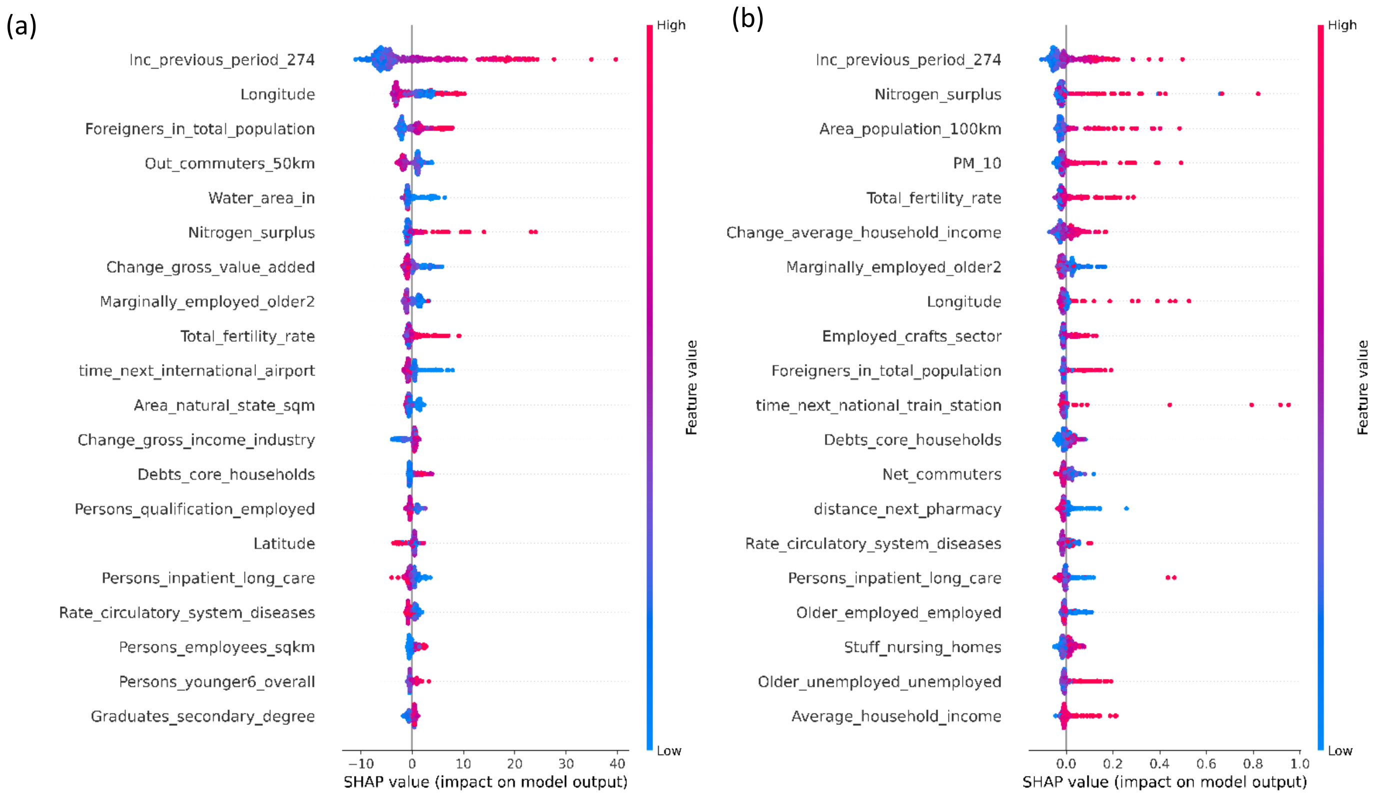

The summary plot combines feature importance with feature effects. Each point on the summary plot is a Shapley value for a feature and a county. The position on the y-axis is determined by the feature and on the x-axis by the Shapley value. The color represents the value of the feature from low to high. Overlapping points are jittered in y-axis direction, to get a sense of the distribution of the Shapley values per feature. The features are ordered according to their importance. E.g. Low values of the age-standardized incidence in the previous period (inc_previous_period_274) are correlated with low values in the age-standardized incidence of the current period (a). High values of longitude (Longitude) are correlated with low values of the age-standardized incidence of the current period (a). High levels of “Nitrogen surplus per agricultural area in kg/ha in 2016” (Nitrogen_surplus) are correlated with high age-standardized death rates in the current period (b). For the exact labels of the features see Supplemental Table 1.

Supplementary Figure S7: Period 2 - SHAP summary plot of the first twenty features (a) age-standardized incidence, (b) age-standardized death rates

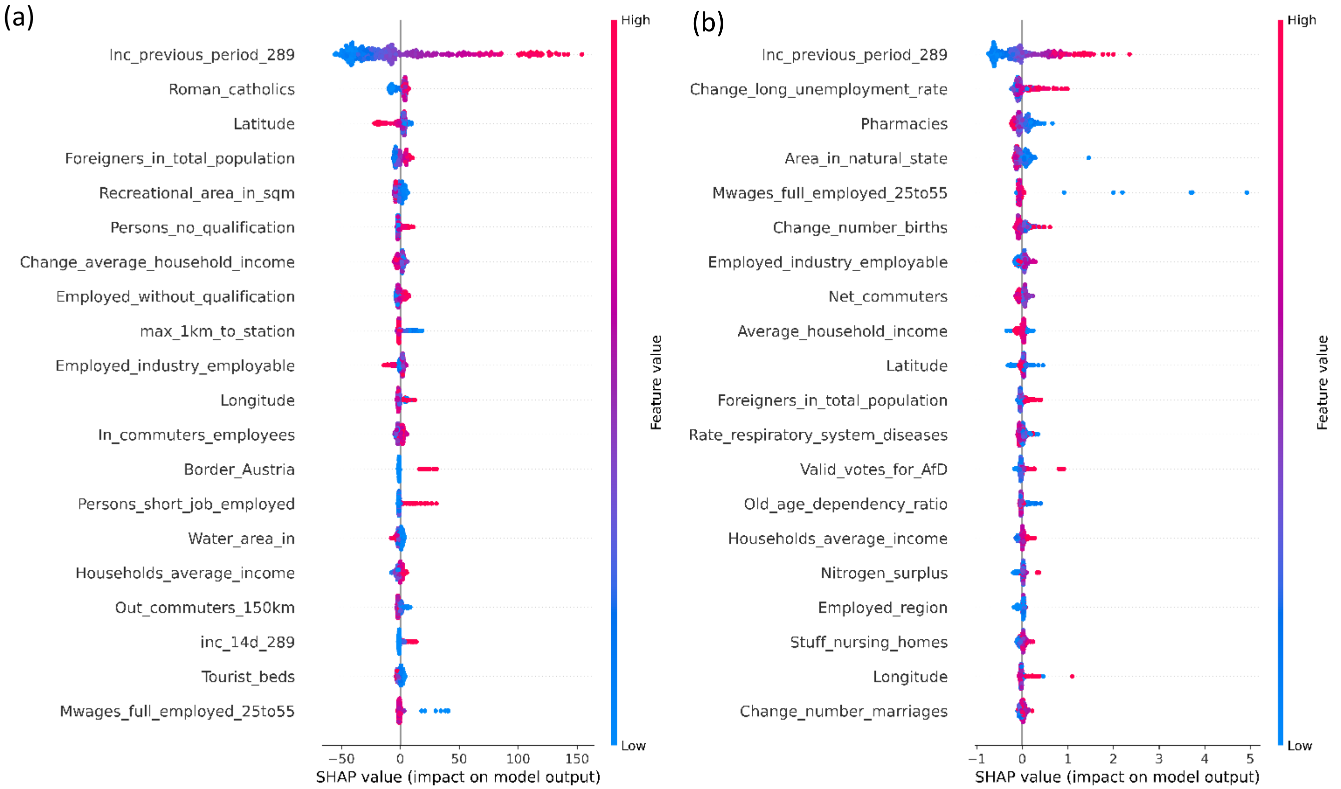

The summary plot combines feature importance with feature effects. Each point on the summary plot is a Shapley value for a feature and a county. The position on the y-axis is determined by the feature and on the x-axis by the Shapley value. The color represents the value of the feature from low to high. Overlapping points are jittered in y-axis direction, to get a sense of the distribution of the Shapley values per feature. The features are ordered according to their importance. E.g. Low values of the age-standardized incidence in the previous period (Inc_previous_period_289) are correlated with low values in the age-standardized incidence (a) and death rate(b) of the current period. A large proportion of people with Roman-Catholic denomination (Roman-catholics) is correlated with low values of the age-standardized incidence of the current period (a). Large "%Changes of the long-term unemployment rate in 2012-2017" (Change_long_unemployment_rate) are correlated with high values of age-standardized death rates in the current period (b). For the exact labels of the features see Supplemental Table 1.

Supplementary Figure S8: Period 3 - SHAP summary plot of the first twenty features (a) age-standardized incidence, (b) age-standardized death rates

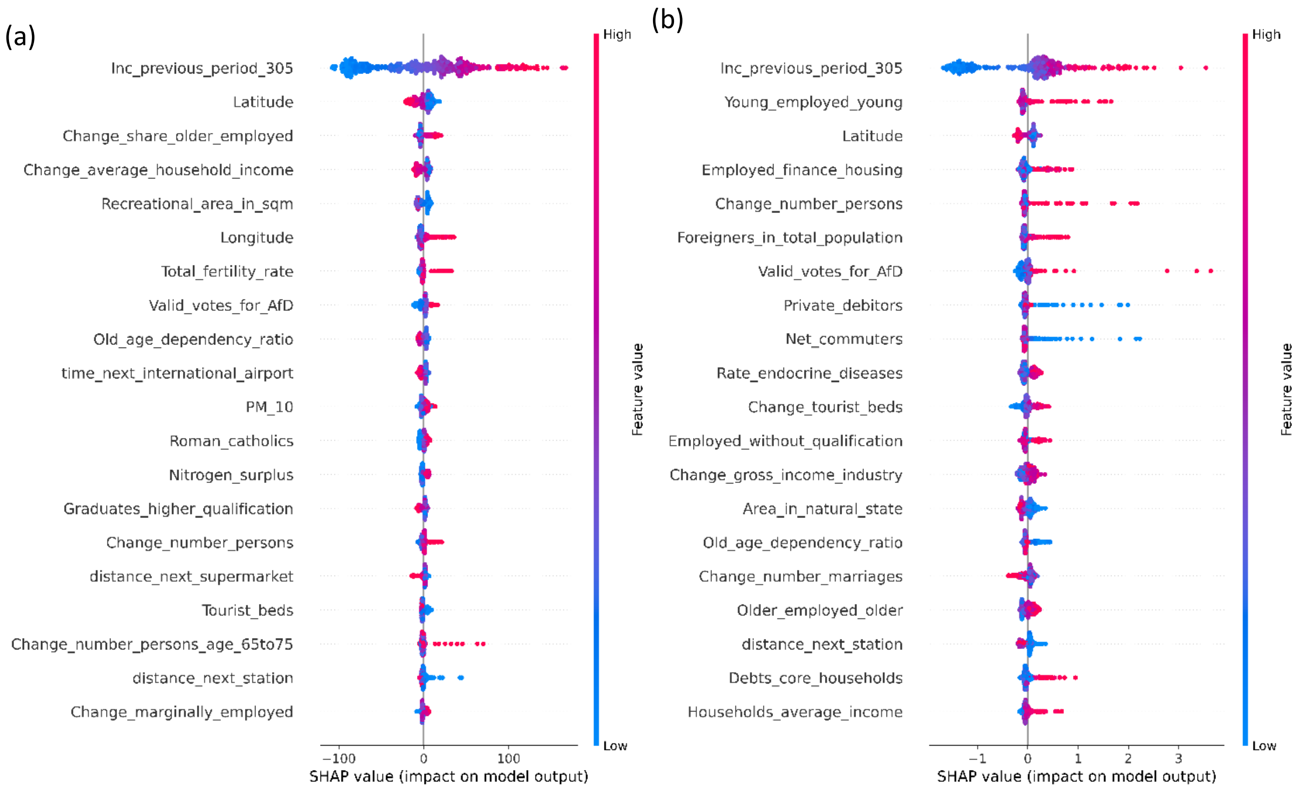

The summary plot combines feature importance with feature effects. Each point on the summary plot is a Shapley value for a feature and a county. The position on the y-axis is determined by the feature and on the x-axis by the Shapley value. The color represents the value of the feature from low to high. Overlapping points are jittered in y-axis direction, to get a sense of the distribution of the Shapley values per feature. The features are ordered according to their importance. E.g. Low values of the age-standardized incidence in the previous period (Inc_previou_period_305) are correlated with low values in the age-standardized incidence (a) and death rate(b) of the current period. A large "%Change of share of older employed persons in all employed persons in 2011-2017" (Change_share_older_employed) is correlated with high values of the age-standardized incidence of the current period (a). A large "%Young employed persons in all young persons (under 26 years) in 2017" (Young_employed_young) is correlated with high values of age-standardized death rates in the current period (b). For the exact labels of the features see Supplemental Table 1.

Supplementary Figure S9: Period 4 - SHAP summary plot of the first twenty features (a) age-standardized incidence, (b) age-standardized death rates

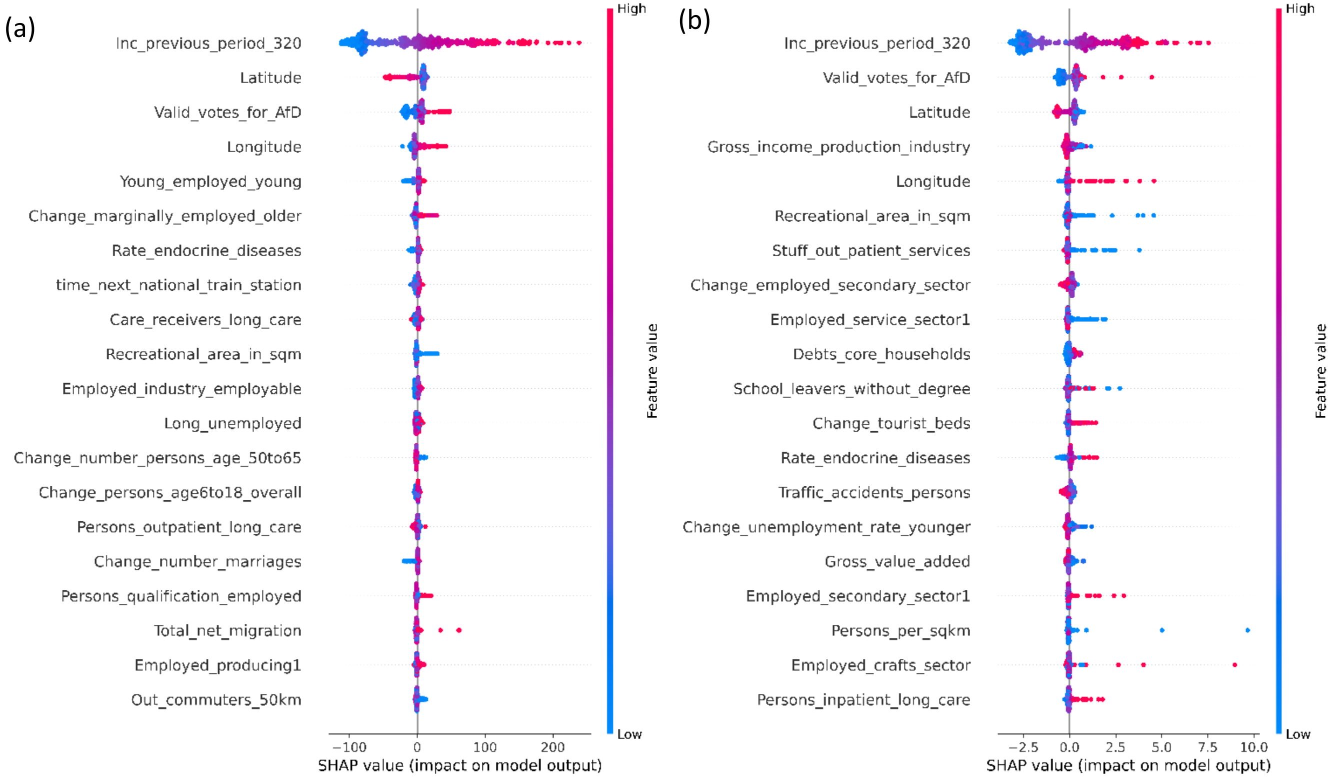

The summary plot combines feature importance with feature effects. Each point on the summary plot is a Shapley value for a featur e and a county. The position on the y-axis is determined by the feature and on the x-axis by the Shapley value. The color represents the value of the feature from low to high. Overlapping points are jittered in y-axis direction, to get a sense of the distribution of the Shapley values per feature. The features are ordered according to their importance. E.g. Low values of the age-standardized incidence in the previous period (Inc_previous_period_320) are correlated with low values in the age-standardized incidence (a) and death rate(b) of the current period. A large “%Valid votes for AfD in all valid votes in 2017” (Valid_votes_for_Afd) is correlated with high values of the age-standardized incidence (a) and death rate (b) of the current period (a). For the labels of the features see Supplemental Table 1.

Supplementary Figure S10: Period 5- SHAP summary plot of the first twenty features (a) age-standardized incidence, (b) age-standardized death rates

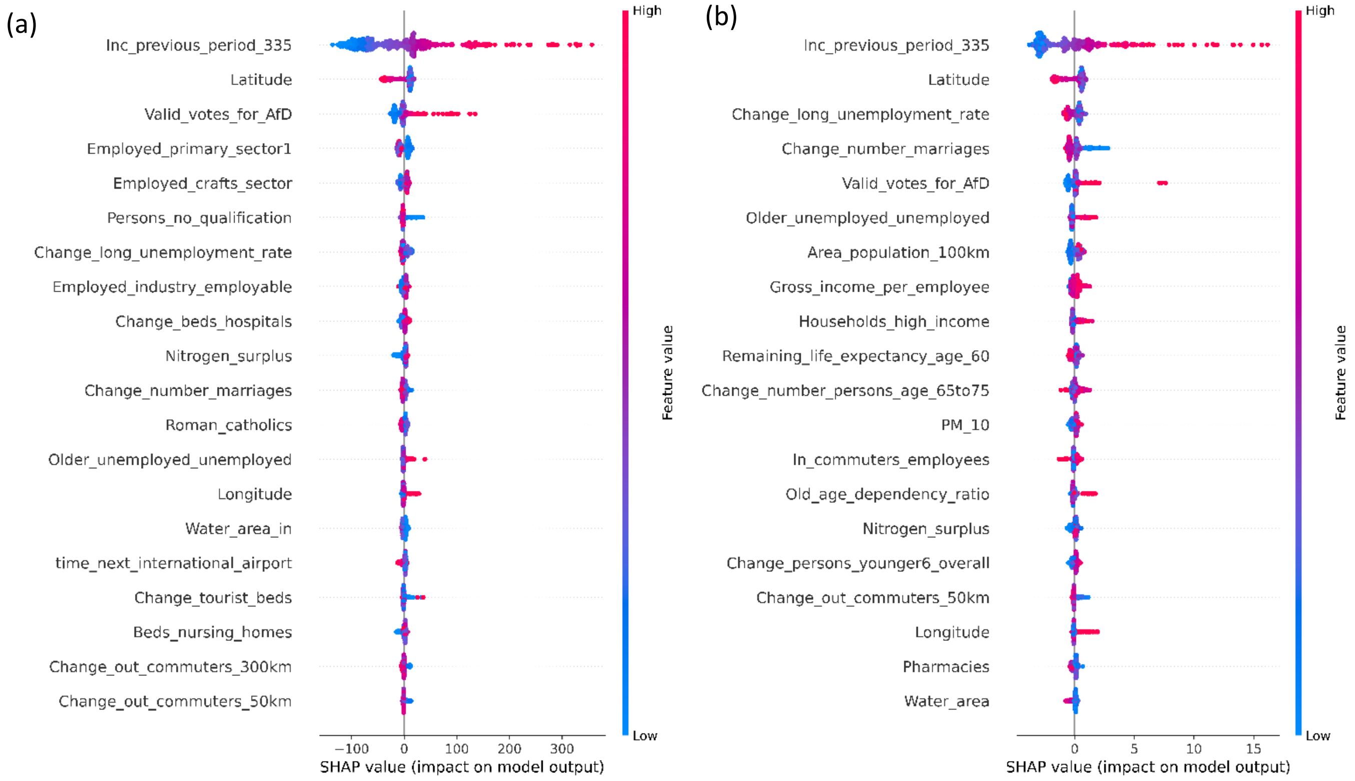

The summary plot combines feature importance with feature effects. Each point on the summary plot is a Shapley value for a fe ature and a county. The position on the y-axis is determined by the feature and on the x-axis by the Shapley value. The color represents the value of the feature from low to high. Overlapping points are jittered in y-axis direction, to get a sense of the distribution of the Shapley values per feature. The features are ordered according to their importance. E.g. Low values of the age-standardized incidence in the previous period (Inc_previ-ous_period_335) are correlated with low values in the age-standardized incidence (a) and death rate(b) of the current period. A large “%Valid votes for AfD in all valid votes in 2017” (Valid_votes_for_AfD) is correlated with high values of the age-standardized incidence (a) and death rate (b) of the current period (a). For the exact labels of the features see Supplemental Table 1.

Table S4: Number of features in the top 10/20 features indicating a positive correlation with high COVID-19 incidence and death rates

Correlation with high incidence	Oct. 01-15				Oct. 16-31				Nov. 01-15				Nov. 16-30				Dec. 01-15			
	IR 10	IR 20	DR 10	DR 20	IR 10	IR 20	DR 10	DR 20	IR 10	IR 20	DR 10	DR 20	IR 10	IR 20	DR 10	DR 20	IR 10	IR 20	DR 10	DR 20
SES high (1)*	2	3	2	3	0	0	1	1	0	0	2	4	0	1	0	3	2	3	3	3
SES low (2)	0	3	1	4	4	8	3	4	2	5	1	5	2	5	4	7	3	4	1	1
Urban/high density (3)	1	3	2	3	0	1	1	2	0	2	0	1	0	0	1	2	0	1	1	4
Rural/low density (4)	1	1	1	2	2	2	1	2	1	2	0	0	2	2	0	1	1	1	0	1
Care need (5)	0	1	0	2	0	0	0	1	0	0	0	0	1	2	1	2	1	2	0	0
Health (6)	1	2	1	2	1	1	1	2	1	1	2	2	2	2	1	2	1	1	2	2
Connect low (7)	0	0	0	1	0	1	1	1	1	2	1	2	0	1	0	0	0	0	0	0
Connect high (8)	1	1	0	0	0	3	0	0	0	0	0	0	0	0	0	0	0	3	0	2
Migration high (9)	2	2	1	1	1	1	0	1	0	0	1	1	0	1	0	0	0	0	0	0
Geography (10)	1	2	1	1	1	2	1	2	2	2	1	1	2	2	2	2	1	2	1	2
Pop. Char.: Values, norms (11)	1	1	0	0	1	1	0	1	1	2	1	1	1	1	1	1	1	2	1	1
Pop. Char.: Age, aging (12)	0	1	1	1	0	0	1	3	2	4	1	3	0	3	0	0	0	1	1	4
Total	10	20	10	20	10	20	10	20	10	20	10	20	10	20	10	20	10	20	10	20

IR 10: Incidence rate first 10 features; IR 20: Incidence rate first 20 features

DR 10: Death rate first 10 features; DR 20: Death rate first 20 features

*: 1=positive SES gradient (SES high); 2=negative SES gradient (SES low); 3=urban/high density gradient (urban); 4=rural/low density gradient (rural); 5=care need stationary/ambulant; 6=health; 7= positive gradient with community's connectedness low (connect low); 8=positive gradient with community's connectedness high (connect high); 9= positive gradient with international migration high (migration high); 10= Geography; 11= population characteristics: Values & norms; 12=population characteristics: Age, aging.
